# The membrane-cytoplasmic linker defines activity of FtsH proteases in *Pseudomonas aeruginosa* clone C

**DOI:** 10.1016/j.jbc.2023.105622

**Published:** 2024-01-03

**Authors:** Gina D. Mawla, Shady M. Kamal, Lian-Ying Cao, Pasi Purhonen, Hans Hebert, Robert T. Sauer, Tania A. Baker, Ute Römling

**Affiliations:** 1 Department of Biology, Massachusetts Institute of Technology, Cambridge, Massachusetts, USA; 2 Department of Microbiology, Tumor and Cell Biology, Karolinska Institutet, Stockholm; Sweden; 3 Department of Biomedical Engineering and Health Systems, KTH Royal Institute of Technology, Huddinge; Sweden

**Keywords:** cytoplasmic linker, essential protease, *Pseudomonas aeruginosa* clone C, ssrA-tag, AAA+ ATPase, M41 protease: periplasmic domain

## Abstract

Pandemic *Pseudomonas aeruginosa* clone C strains encode two inner-membrane associated ATP-dependent FtsH proteases. Pa*ftsH1* is located on the core genome and supports cell growth and intrinsic antibiotic resistance, whereas Pa*ftsH2*, a xenolog acquired through horizontal gene transfer from a distantly related species, is unable to functionally replace Pa*ftsH1*. We show that purified PaFtsH2 degrades fewer substrates than PaFtsH1. Replacing the 31-amino acid–extended linker region of PaFtsH2 spanning from the C-terminal end of the transmembrane helix-2 to the first seven highly divergent residues of the cytosolic AAA+ ATPase module with the corresponding region of PaFtsH1 improves hybrid-enzyme substrate processing *in vitro* and enables PaFtsH2 to substitute for PaFtsH1 *in vivo*. Electron microscopy indicates that the identity of this linker sequence influences FtsH flexibility. We find membrane-cytoplasmic (MC) linker regions of PaFtsH1 characteristically glycine-rich compared to those from FtsH2. Consequently, introducing three glycines into the membrane-proximal end of PaFtsH2’s MC linker is sufficient to elevate its activity *in vitro* and *in vivo*. Our findings establish that the efficiency of substrate processing by the two PaFtsH isoforms depends on MC linker identity and suggest that greater linker flexibility and/or length allows FtsH to degrade a wider spectrum of substrates. As PaFtsH2 homologs occur across bacterial phyla, we hypothesize that FtsH2 is a latent enzyme but may recognize specific substrates or is activated in specific contexts or biological niches. The identity of such linkers might thus play a more determinative role in the functionality of and physiological impact by FtsH proteases than previously thought.

FtsH, a membrane-bound protease and member of the AAA+ (ATPases associated with diverse cellular activities) superfamily of ATPases, is widespread in prokaryotes and in eukaryotic organelles (1, 2, 3). Although some bacteria encode five distinct classes of AAA+ proteases, FtsH is typically the only protease that is biologically essential, is the most phylogenetically conserved, and is the only inner membrane–associated AAA+ protease in most Gram-negative bacteria (4). FtsH is an essential modulator of lipid metabolism in *Escherichia coli* and is important for growth, virulence, production of secondary metabolites, biofilm formation, and formation of persister cells in *Pseudomonas aeruginosa* and many other bacteria (5, 6, 7, 8, 9, 10). Model bacteria, including *P. aeruginosa*, typically encode just one FtsH enzyme. The pandemic clone C group of *P. aeruginosa* strains, found at high prevalence in acute and chronic infections and the aquatic habitat (11, 12), however, is an example of a distinct group of organisms that have undergone recent ‘*ftsH*-gene expansion’ as it carries a second FtsH proteinase introduced by horizontal transfer (5, 13).

Like other AAA+ proteases, FtsH functions as a hexamer with an axial channel that serves as a conduit for polypeptide translocation into a degradation chamber. A prototypical FtsH subunit begins with a short N-terminal cytoplasmic segment, followed by a transmembrane helix (TM1), a periplasmic domain, another transmembrane helix (TM2), a cytoplasmic AAA+ substrate unfolding/translocation module, and a cytoplasmic M41-family peptidase domain (Figs. 1*A* and S1). A cytosolic segment of ∼25-residues connects TM2 with the AAA+ module and hereafter is called the MC or membrane-cytosolic linker. As FtsH is membrane-tethered, it has been suggested that this linker may help gate substrate entry *via* the conserved FVG pore (3). Together, these distinct regions of FtsH are required for its function (14, 15, 16, 17, 18, 19, 20).

**Figure 1:**
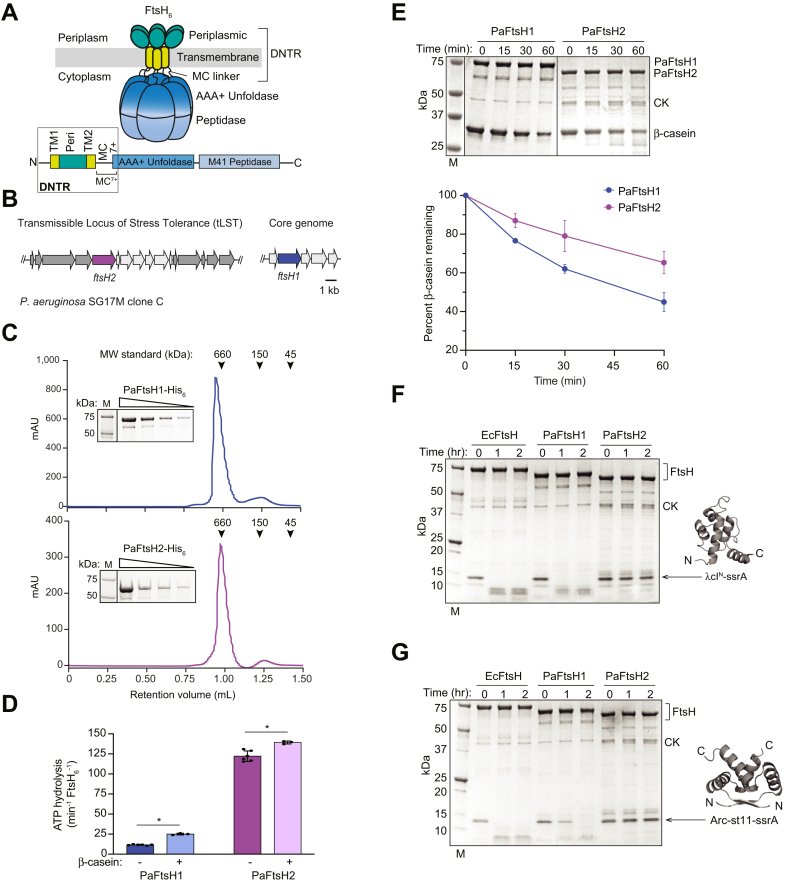
**Purified hexameric PaFtsH1 and PaFtsH2 have distinct biochemical activities *in vitro*.***A*, schematic (*top*) and linear representation (*bottom*) of a prototypical hexameric FtsH enzyme in a bacterial cell with key regions labeled. The DNTR spans the N-terminus, TM1, periplasmic domain, TM2, MC linker, and the first seven amino acids in the AAA+ ATPase module (MC^7+^). *B*, schematic of genetic loci of *PaftsH* genes in *Pseudomonas aeruginosa* SG17M clone C strain. PaFtsH1 is encoded on the core genome of SG17M (*blue*), and PaFtsH2 (*purple*) is encoded on a horizontally acquired genomic island termed the transmissible locus of stress tolerance (tLST) which is distinctive in SG17M clone C. Names of genes in the vicinity of *ftsH1* and *ftsH2* are labeled in Fig. S2*A*. In *dark gray*, *P. aeruginosa* SG17M specific tLST genes; *light gray*, additional tLST genes present in other tLST classes. *C*, molecular weight determination of PaFtsH1 and PaFtsH2 purified from *Escherichia coli*. Purified PaFtsH1 and PaFtsH2 were run on an analytical Superdex 200 3.2/3.0 gel filtration column (*left*) and separated by a 4 to 20% gradient SDS-PAGE in a two-fold dilution series (insets). Molecular weights of PaFtsH enzymes were consistent with hexamer formation plus ∼3.5 NP-40 micelles per PaFtsH1-6xHis hexamer (PaFtsH1_6_-6xHis: 743,373 Da; one NP-40 micelle: ∼90 Da) and ∼2.7 NP-40 micelles per PaFtsH2-6xHis hexamer (PaFtsH2-6xHis: 659,792 Da). Theoretical molecular weights of 6xHis-tagged PaFtsH monomers: PaFtsH1-6xHis: 70,886 Da; PaFtsH2-6xHis: 69,521 Da. *D*, hydrolysis of ATP (5 mM) by PaFtsH1 (*blue* bars) or PaFtsH2 (*purple* bars) (0.43 μM) in the presence or absence of the substrate β-casein (40 μM) at 40 °C. ATP hydrolysis rates by PaFtsH1 and PaFtsH2 increased in the presence of β-casein (two-tailed Mann-Whitney U test (∗*p* < 0.05)). The rate of ATP hydrolysis by β-casein in the absence of enzyme was negligible (0.0032 ± 0.0004 ATP min^−1^). *E*, Forty micromolar β-casein was incubated at 40 °C with PaFtsH1 or PaFtsH2 (0.43 μM), and degradation kinetics were monitored by a 4 to 20% gradient SDS-PAGE. Data points are averages of three independent replicates ± SD (*bottom*). M: Molecular weight marker taken from the PaFtsH2 casein degradation gel. *F*, the N-terminal domain of phage λ repressor (λcI^N^) with a C-terminal ssrA degron tag (λcI^N^-ssrA; 15 μM) was incubated at 40 °C with EcFtsH, PaFtsH1, or PaFtsH2 (3.04 μM). λcI^N^-ssrA PDB: 1LM (82). M: Molecular weight marker. *G*, SsrA degron-tagged Arc repressor-(6 x H)KNQHE (Arc-st11-ssrA; 15 μM) was incubated at 40 °C with EcFtsH, PaFtsH1, or PaFtsH2 (3.53 μM). Arc-st11-ssrA PDB: 1ARR. M: Molecular weight marker. *D*–*G*, all reactions used C-terminal 6xHis-tagged proteins. *E*-*G*, all reactions contained 5 mM ATP and a creatine kinase (CK)-based ATP regeneration system (83). DNTR, diverse N-terminal region; MC, membrane-cytoplasmic; MC^7+^, membrane-cytoplasmic linker plus seven C-terminal amino acids into the AAA+ ATPase module boundary; MW, Molecular weight; M, Molecular weight marker; mAU, milli-Absorbance Unit; MC^7+^, membrane-cytoplasmic plus next seven C-terminal amino acids; Peri, periplasmic domain; TM1, transmembrane helix 1; TM2, transmembrane helix 2.

FtsH recognizes protein substrates by binding to N- or C-terminal degrons, with the amino acid sequences determining the degradation rate, and, less commonly, to internal disordered regions (10, 14, 21, 22). One group of FtsH substrates includes incomplete proteins tagged with a C-terminal ssrA degron, a short predominantly nonpolar peptide sequence attached to nascent chains *via* its transfer-messenger RNA–mediated stalled ribosome rescue (21, 23, 24). FtsH participates in membrane-protein quality control by degrading misassembled and misfolded inner membrane proteins and regulates proteotoxic stress by controlling intracellular levels of the RpoH/σ^32^ heat-shock transcription factor (5, 25, 26, 27, 28, 29). In some cases, adaptor proteins influence the degradation efficiency and specificity of FtsH proteases. For example, in *E. coli*, the heat shock transcription factor RpoH requires the co-chaperone DnaK and the signal recognition particle to be efficiently processed by core genome FtsH (30, 31, 32). On the other hand, the periplasmic membrane proteins, HflK and HflC, interact with FtsH (33) and negatively regulate proteolysis of the protein translocase subunit SecY (28) and the λ transcriptional regulator cII (34). Other adaptor proteins of FtsH influence degradation of LpxC, the first committed enzyme in lipopolysaccharide biosynthesis, and processing of the polypeptide toxin Colicin D (8, 35).

As all clone C strains, the virulent aquatic isolate of *P. aeruginosa* SG17M carries a transmissible locus of stress tolerance (tLST) important for survival under multiple stress conditions that encodes a xenolog of FtsH called PaFtsH2 ((Figs. 1*B* and S2*A*); (12, 13)). We previously found that deletion of the core genome FtsH copy, Pa*ftsH1*, causes a substantial growth deficiency and affects multiple other phenotypes including secondary metabolite secretion and antimicrobial resistance, whereas PaFtsH2 appears to act primarily as a low-level backup enzyme under the growth and stress conditions tested (5, 36). Here, we investigate the molecular basis of the differential activities and impacts of PaFtsH1 and PaFtsH2. *In vivo* observations indicated that the N-terminal region of the PaFtsH1 and PaFtsH2 homologs, which include TM1, TM2, the periplasmic domain, the MC linker, and the first seven amino acids of the AAA+ ATPase module, is necessary and sufficient to determine function of the two distinct PaFtsH enzymes *in vivo*. Using phenotypic and biochemical assays, we identified a 32-amino acid stretch composed of the MC linker (25-amino acids long) and first seven resides of the AAA+ ATPase module (referred to herein as the membrane-cytoplasmic plus next seven C-terminal amino acids (MC^7+^) linker region) as a determinative element of PaFtsH function. Even more, inserting just three glycines into the PaFtsH2 linker to mimic the glycine-rich feature of the PaFtsH1 sequence results in a ‘gain-of-function’ phenotype. Notably, purified PaFtsH2 hybrids carrying PaFtsH1-like MC linker alterations gain proteolytic activity against model substrates *in vitro*. We propose a model that explains how the composition, length, and flexibility of the MC linker could modulate PaFtsH enzyme function by affecting the position and mobility of the cytoplasmic AAA+ protease with respect to the plane of the membrane and control the accessibility of specific substrates to the AAA+ axial channel during the initiation of efficient proteolysis.

## Results

### The N-terminal regions of PaFtsH1 and PaFtsH2 are highly divergent

To identify the molecular mechanism of the differential activity of PaFtsH2 compared to PaFtsH1 observed *in vivo* (5), we first assessed the amino acid sequence conservation of the individual domains. The overall sequence identity between PaFtsH1 and PaFtsH2 is 45%, with the most conserved regions being the AAA+ module and the Zn^2+^-dependent M41 peptidase domain (54% identity). Signature motifs for substrate processing: the pore motif FVG for substrate entry and motifs required for catalytic activities of the AAA+ module (Walker A and Walker B motifs) and the M41 peptidase domain (Zn^2+^-binding motif and catalytic base) are completely conserved in PaFtsH2 and PaFtsH1 for both domains (Figs. 1*A* and S1). Presence of these and other consensus residues therefore suggests that any functional differences between these two multidomain enzymes would not be attributed to differences in these motifs. We found, however, the N-terminal ∼150 amino acids to be most divergent (∼24% identity) (Fig. S1). We refer to this region from the N terminus through the first β-strand of the AAA+ module as the divergent N-terminal region (DNTR) (Figs. 1*A* and S1).

### Purified PaFtsH1 and PaFtsH2 assemble as hexameric ATPases and proteases

To compare the intrinsic biochemical activities of PaFtsH1 and PaFtsH2, we overexpressed each His-tagged enzyme in *E. coli* and purified them from cell membranes according to a protocol previously established for *E. coli* FtsH (24). Each enzyme eluted from an analytical size-exclusion column as a single peak, with a molecular weight expected for a hexamer bound to detergent micelles (Fig. 1*C*). Both enzymes were active ATPases, with ATP hydrolysis by PaFtsH2 being ∼nine-fold faster than by PaFtsH1 under standard conditions (Figs. 1*D* and S2*B*) despite being the ‘silent’ gene product, a latent enzyme with no explicit proteolytic activity *in vivo* and in most *in vitro* instances ((5); and as described below). The temperature optimum for ATPase activity was 45 °C and 55 °C for PaFtsH1 and PaFtsH2, respectively, which is above the standard and maximum growth temperature, 30 °C-37 °C and 42 °C, respectively, for *P. aeruginosa*. Addition of β-casein, a commonly used protease substrate with a molten-globule structure, stimulated the PaFtsH1 ATPase activity about two-fold but only marginally stimulated that of PaFtsH2 (Fig. 1*D*). Both enzymes effectively degraded β-casein with PaFtsH1 degradation rate being slightly (∼two-fold) faster than PaFtsH2 (Fig. 1*E*). Thus, purified PaFtsH1 and PaFtsH2 are active as ATPases and proteases *in vitro*.

### PaFtsH2 fails to degrade ssrA-tagged proteins

*E. coli* FtsH degrades protein substrates with C-terminal ssrA tags (Fig. 1, *F* and *G*; (24, 37)). SsrA is a well-recognized autonomous degron that can be added to essentially any protein, rendering it a substrate. In the case of SsrA-tagged peptides subjected to degradation by FtsH protease, additional cofactors that aid processing do not seem to be required. Thus, we tested PaFtsH1 and PaFtsH2 degradation of λcI^N^-ssrA, the N-terminal DNA-binding domain of the phage λ repressor cI (λcI^N^) with the *E. coli* 11-amino acid ssrA tag (-AANDENYALAA) (38). As assayed by an 4 to 20% SDS-PAGE gel, PaFtsH1 degraded this substrate over the course of ∼1 h, whereas PaFtsH2 was essentially inactive in degradation of this substrate (Fig. 1*F*). Notably, PaFtsH1 did not degrade λcI^N^ lacking the ssrA degron (Fig. S3*A*). PaFtsH1, but not PaFtsH2, also degraded the Podoviridae family phage P22 Arc repressor model protein substrate Arc-st11 containing a *P. aeruginosa* ssrA tag (AANDDNYALAA; Fig. 1*G*). Note that throughout this work, we found that PaFtsH1 efficiently recognized both the *P. aeruginosa* sequence*-*specific ssrA-tag (-AANDDNYALAA) and the *E. coli* ssrA sequence (-AANDENYALAA), which differ by just one amino acid.

### PaFtsH1 and PaFtsH2 have similar peptide-binding profiles

**Figure 2:**
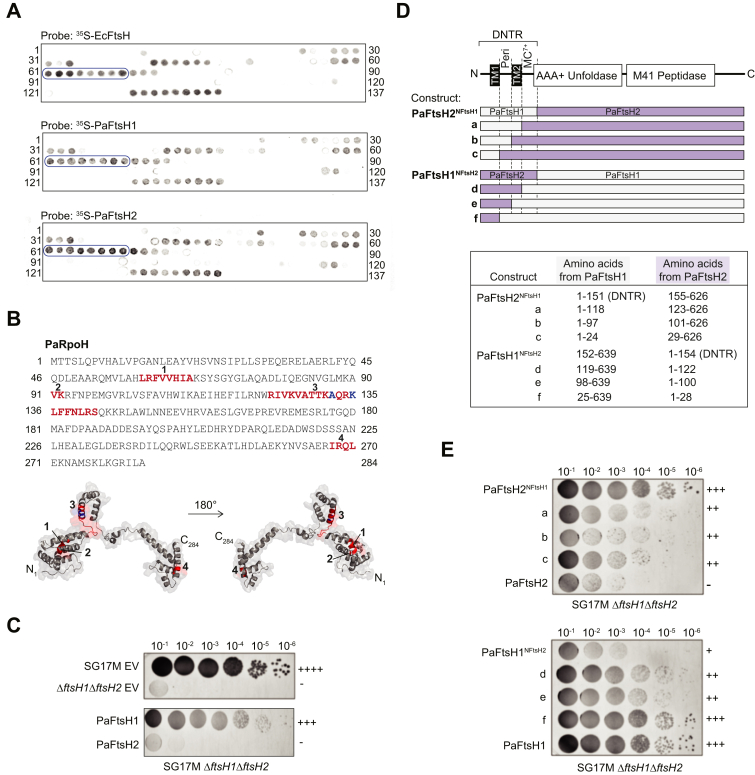
**PaFtsH1 and PaFtsH2 have similar *in vitro* peptide-binding profiles and differentially affect *in vivo* colony size.***A*, peptide array of the RpoH sequence from *Pseudomonas aeruginosa* (PaRpoH; P42378|RpoH_PSEAE; 1–284, sliding window of 12 amino acids with a step size of two amino acids towards the C-terminus with each spot) probed with 1 μM ^35^S-EcFtsH (*top*), ^35^S-PaFtsH1 (*middle*), or ^35^S-PaFtsH2 (*bottom*) in the presence of 1.25 mM ATPγS. Peptide sequences corresponding to each spot are listed in Table S1. *Circled* spots indicate peptides containing residues A132 and/or K135, which were previously shown to be important for *in vivo Escherichia coli* FtsH (EcFtsH) binding to RpoH (conserved RpoH residues from *E. coli* are A131 and K134; (39, 40)). *B*, amino acid sequence of PaRpoH (*top*). Residues in *red* indicate regions bound by ^35^S-EcFtsH, ^35^S-PaFtsH1, ^35^S-PaFtsH2. Residues in *blue* correspond to A132 and K135 (see *panel A* legend). FtsH-bound sequences identified by peptide blotting are mapped on the predicted PaRpoH structure (*bottom*; (72)) and numbered as on the sequence above. *C*, colony sizes of wild-type *P. aeruginosa* SG17M, the SG17M Δ*ftsH1*Δ*ftsH2* double deletion and SG17M Δ*ftsH1*Δ*ftsH2* strains complemented with either an empty pJN105 expression vector (EV) or pJN105-derived expression of PaFtsH1 or PaFtsH2. Pa*ftsH1* and Pa*ftsH2* genes had been cloned into the expression vector pJN105 under the translational regulation of the Pa*ftsH2* Shine Dalgarno (SD) sequence. “+” and “-” scoring system relates the qualitative degree of colony growth of a particular strain genotype relative to that of SG17M EV (++++; wild-type colony growth) or SG17M Δ*ftsH1*Δ*ftsH2* (-; very poor colony growth). For (*C* and *E*), numbers along the *top* refer to the serial dilution factor for plating cells. *D*, schematic representation of PaFtsH1 and PaFtsH2 hybrid protein constructs. Regions corresponding to PaFtsH1 and PaFtsH2 identity are depicted in *gray* and *purple*, respectively (*top*). Designation of protein variants and regions derived from PaFtsH1 and/or PaFtsH2 that make up the respective FtsH hybrid variants are indicated in the table (*bottom*). *E*, colony size of the *P. aeruginosa* SG17M Δ*ftsH1*Δ*ftsH2* double deletion strain complemented with Pa*ftsH1* and Pa*ftsH2* hybrid constructs as introduced in *panel D*. “+” and “-” scoring system is the same as in (*C*). DNTR, Diverse N-terminal region; MC^7+^, membrane-cytoplasmic linker plus seven C-terminal amino acids into the AAA+ ATPase module boundary; TM1, Transmembrane helix 1; TM2, Transmembrane helix 2.

As degrons are often unstructured peptide loops exposed on a protein surface, measuring binding of an AAA+ M41 protease to immobilized peptide arrays can be a useful method for identifying and characterizing degron sequences of substrates. However, these binding experiments must be followed up by functional assays to establish the role of identified sequences in protease recognition of the intact protein. To rapidly investigate if PaFtsH1 and PaFtsH2 had similar or distinct peptide-binding properties, we synthesized arrays of 12-residue peptides from known or probable FtsH substrates from *P. aeruginosa*. Known PaFtsH1 substrates include RpoH and the phenazine biosynthesis enzyme PhzC1/2 (identical), and PaFtsH1 or PaFtsH2 candidate substrates include phosphoglucosamine mutase GlmM, the septum-site determining protein MinD, and the signal peptidase LepB1, respectively (5, 29). Subsequently, peptide arrays were probed with ^35^S-PaFtsH1 and ^35^S-PaFtsH2 (Figs. 2, *A* and *B*, S4, and Table S1). Radiolabeled PaFtsH1 and PaFtsH2 bound to these arrays with very similar profiles, indicating that both enzymes interact with the immobilized linear peptides in the same manner. Note that this approach revealed strong binding of *E. coli* FtsH and both *P. aeruginosa* enzymes also to a sequence in RpoH (region 3, Fig. 2, *A* and *B*) that is known to be important for degradation in *E. coli* (39, 40). These findings indicate that at least some of the protein–protein interactions identified here are involved in substrate recognition by these enzymes and that the linear epitopes of the substrates are recognized in an identical way by the two enzymes.

### The C-terminal region of the DNTR determines *in vivo* impact

**Figure 3:**
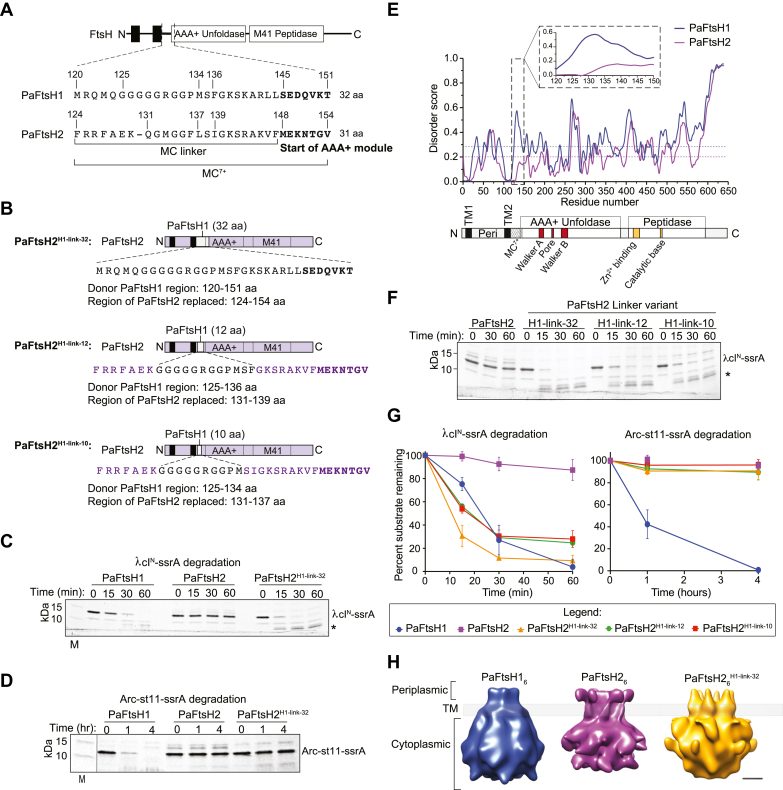
**The membrane-cytoplasmic MC**^**7+**^**linker of PaFtsH1 activates degradation by PaFtsH2 *in vitro*.***A*, linear map of a model FtsH enzyme with a zoom-in on MC^7+^ linker sequences from PaFtsH1 (32 amino acids) and PaFtsH2 (31 amino acids). Bolded residues denote the start (N-terminal side) of the respective PaFtsH1 and PaFtsH2 AAA+ ATPase modules. *B*, schematic of PaFtsH2 MC^7+^ and MC linker chimera constructs: PaFtsH2^H1-link-32^, PaFtsH2^H1-link-12^, PaFtsH2^H1-link-10^. Regions in *gray* represent residues of donor PaFtsH1 identity on an otherwise PaFtsH2 host (*purple*). The number of amino acids derived from PaFtsH1 are indicated in the superscript of the variant name. *Black filled rectangles* represent TM domains. *C*, degradation of λcI^N^-ssrA substrate (15 μM) by PaFtsH1, PaFtsH2, and PaFtsH2^H1-link-32^ (3.04 μM) as described in Figure 1*F*. Bands corresponding to full-length λcI^N^-ssrA substrate and a ∼5.7 kDa intermediate degradation product (denoted by *asterisk*) are indicated. *D*, degradation of Arc-st11-ssrA (15 μM) was monitored in the presence of PaFtsH1, PaFtsH2, and PaFtsH2^H1-link-32^ (3.53 μM) as described in Figure 1*G*. *E*, structural disorder prediction profiles of PaFtsH1 (*blue*) and PaFtsH2 (*purple*) as anticipated by the IUPred algorithm (74). *Dotted lines* indicate the average disorder scores for residues 1 to 590 of PaFtsH1 (*blue*, 0.29) and PaFtsH2 (*purple*, 0.20). Inset shows a zoom-in of the MC^7+^ linker regions. Bottom schematic is a linear model of an FtsH enzyme with relevant structural and functional regions noted on the diagram. *F*, degradation of λcI^N^-ssrA substrate (15 μM) was monitored in the presence of PaFtsH1, PaFtsH2, and PaFtsH2^H1-link-32^ (3.04 μM) as described in Figure 1*F*. *G*, quantification of full-length substrate remaining over reaction time courses. Error bars represent SD of experiments performed in triplicate. Representative degradation kinetics as monitored by SDS-PAGE by PaFtsH1 and PaFtsH2 are shown in Figure 1, *E* and *F*. *H*, 3D-reconstructions from negative stain TEM of PaFtsH1, PaFtsH2, and PaFtsH2^H1-link-32^. Scale bar represents 3 nm. Periplasmic, transmembrane (TM), and cytoplasmic domains are noted on the figure. MC^7+^, membrane-cytoplasmic plus next seven C-terminal amino acids; Peri, Periplasmic domain; TM1, Transmembrane domain 1; TM2, Transmembrane domain 2.

We have reported previously that although both PaFtsH1 and PaFtsH2 are produced in exponential phase in rich and nutrient-poor medium (with PaFtsH2 even showing enhanced production in stationary phase, while PaFtsH1 production highly diminished), PaFtsH1 plays an almost exclusive role in supporting robust growth, as measured by colony size and growth rate (5). Figure 2*C* recalls that leaky expression of plasmid-based Pa*ftsH1* but not Pa*ftsH2* under the translational control of the Pa*ftsH2* Shine-Dalgarno sequence rescues the tiny colony size phenotype of SG17M Δ*ftsH1*Δ*ftsH2*. Of note, plasmid-based production of any of the two proteins does not affect plating efficiency as equal dilutions starting from the same OD_600_ yielded the same number of colonies irrespectively of colony size and growth rate (5). We thus used colony size as the phenotypic assay to estimate alterations in PaFtsH1-linked functionality as neither PaFtsH1 nor PaFtsH2 protein production are directly correlated with functionality (5). To determine if DNTR, the most distinct region between these two proteins, determines functionality under these experimental conditions, we expressed constructs containing residues 1 to 151 of PaFtsH1 (PaFtsH1 DNTR) appended to the “body” of PaFtsH2 (PaFtsH2^NFtsH1^) or residues 1 to 154 of PaFtsH2 (PaFtsH2 DNTR) attached to “body” of PaFtsH1 (PaFtsH1^NFtsH2^) under translational control of an identical Pa*ftsH2* Shine-Dalgarno sequence in SG17M Δ*ftsH1*Δ*ftsH2*. Notably, PaFtsH2^NFtsH1^ rescued the colony size defect whereas PaFtsH1^NFtsH2^ did not (Fig. 2, *C*–*E*), indicating that the DTNR of *ftsH1* can function with the AAA+ module and M41 peptidase of Pa*ftsH2* to support robust growth, while the DTNR of PaFtsH2 even in combination with the body of PaFtsH1 does not promote functionality. To identify the minimal regions of the PaFtsH1 DTNR that contribute most strongly to PaFtsH functionality, we constructed and tested additional PaFtsH1 and PaFtsH2 chimeras with iteratively swapped DTNR segments informed by the predicted boundaries for the two transmembrane helices and the periplasmic domains (Figs. 2, *D* and *E* and S1; (5)). We found that for PaFtsH2, restoration of colony size was largely determined by an amino acid stretch of PaFtsH1 that included the MC linker and the N-terminal seven residues of β-strand 1 in the AAA+ module (see especially Fig. 2*E*, construct a compared to PaFtsH2^NFtsH1^). On the other hand, the equivalent amino acid stretch from PaFtsH2 abolished the restoration of colony size by PaFtsH1 (Fig. 2*E*, construct d compared to PaFtsH1^NFtsH2^). We subsequently defined the 32-amino acid long sequence of PaFtsH1 from Met^120^ to Thr^151^ and the corresponding 31-amino acid long sequence of PaFtsH2 from Phe^124^ to Val^154^ as the MC^7+^ linker (Figs. 3*A* and S1).

### MC^7+^ sequences from PaFtsH1 activate degradation by PaFtsH2

To unambiguously assess the contribution of the MC^7+^ to functionality, we constructed, expressed, and purified a variant called PaFtsH2^H1-link-32^ containing the 32-residue MC^7+^ region from PaFtsH1 with all other amino acid sequences derived from PaFtsH2 (Fig. 3, *A* and *B*). Strikingly, PaFtsH2^H1-link-32^ degraded λcI^N^-ssrA, whereas the parental PaFtsH2 protease did not. PaFtsH2^H1-link-32^ also degraded the λcI^N^-ssrA substrate at a rate faster than PaFtsH1 in an ATP- and ssrA tag-dependent manner (Figs. 3*C* and S3, *A* and *B*). However, PaFtsH2^H1-link-32^, like its PaFtsH2 parent, was unable to degrade Arc-st11-ssrA (Fig. 3*D*), suggesting that the MC^7+^ region from PaFtsH1 is not directly involved in recognition of the ssrA tag or that such recognition is highly dependent on features of the attached protein. PaFtsH2^H1-link-32^ degradation of λcI^N^-ssrA generated a ∼5.7 kDa product (marked by asterisks in Figs. 3, *C* and *F* and S3, *A* and *B*) that mass spectrometry identified as residues 1 to 51 of λcI^N^-ssrA, as expected if proteolysis initiates from the C-terminal ssrA degron of this substrate (Fig. S3*C*). The presence of this degradation product could represent a difference in peptide-cleavage specificity within the protease chambers of PaFtsH1 and PaFtsH2 or a difference in substrate processing by the AAA+ modules of these enzymes. Indeed, it has been observed that FtsH can perform limited proteolytic cleavage (35).

We continued with the linker dissection approach, constructed hybrid open reading frames, and purified corresponding enzymes containing a hybrid PaFtsH2 with just 12 or 10 residues of the MC linker of PaFtsH1 replacing a corresponding PaFtsH2 linker sequence (Fig. 3*B*). These amino acid sequences encompassed the characteristic glycine-rich part of the PaFtsH1 linker from Gly125 to Met134/Phe136, respectively. Importantly, unlike the parental PaFtsH2, both chimeras degraded λcI^N^-ssrA like PaFtsH2^H1-link-32^ with a not substantially slower initial kinetics but again not to completion (Figs. 3, *F* and *G* and S3*A*). The MC^7+^ linker of PaFtsH1 is predicted by computational methods to be more disordered/flexible than that of PaFtsH2, as are the linker segments containing just 12 or 10 PaFtsH1 residues (Figs. 3*E*, S3*D*, and S5).

### The MC^7+^ linker appears to contribute to conformational flexibility

To investigate whether the functional differences we observed with the different MC linkers are reflected in differences in enzyme structure/dynamics, we imaged detergent-solubilized PaFtsH1, PaFtsH2, and the most active hybrid protein PaFtsH2^H1-link-32^ by electron microscopy and negative staining, resulting in fields of particles representing single hexamers (Fig. S6*A*). Reconstructed 3D-maps using six-fold symmetry produced distinct ∼22-Å resolution density maps for the three enzymes (Fig. 3*H*). Each map contained obvious density for the large cytoplasmic domains and weaker density for the periplasmic domain, whereas the transmembrane helices were obscured by detergent and not well-visualized (Fig. S6*B*).

Although PaFtsH1 and PaFtsH2 showed cone-shaped appearances, more defined-shape features were visible for PaFtsH2, including well-defined domain-like shapes in the periplasmic ring and clear delineation between the AAA+ ATPase and peptidase segments of the cytoplasmic regions (Fig. 3*H*). We interpret these structural differences as an indication of PaFtsH2’s relative conformational homogeneity and rigidity, whereas PaFtsH1 may adopt more, slightly different, conformations and thus “blur” structural features. These observations are consistent with the hypothesis that the shorter, stiffer MC^7+^ linker in PaFtsH2 confines its movements, potentially hampering substrate engagement and/or unfolding. Interestingly, the structural definition in the PaFtsH2^H1-link-32^ variant was an intermediate between those of PaFtsH1 and PaFtsH2. Especially noticeable in PaFtsH2^H1-link-32^ was the loss of cytoplasmic-ring definition compared to parental PaFtsH2. These results also support the notion that the PaFtsH1 MC7+ linker imparts greater ability to “explore” more conformations than the PaFtsH2 linker.

The PaFtsH2 structure was more similar to a recent cryo-EM structure of hyperthermophilic *Aquifex aeolicus* FtsH ((3); EMD-11161) than the other structures, probably because it had the most well-defined periplasmic and cytoplasmic domains. Of note, although phylogenetically more similar to PaFtsH1 (46% *vs.* 42% identity), AaFtsH is predicted to possess a more rigid linker than PaFtsH1 with three glycine residues (Fig. S1). The crystal structure of the *A. aeolicus* cytoplasmic domain of FtsH (PDB 4WW0; (41)) also fit best with PaFtsH2, although the AAA+ modules and peptidase domains could be acceptably fit in each of the three density maps (Fig. S6*B*).

### PaFtsH1 MC^7+^ linker length determines complementation strength by PaFtsH1-PaFtsH2 chimeras

**Figure 4:**
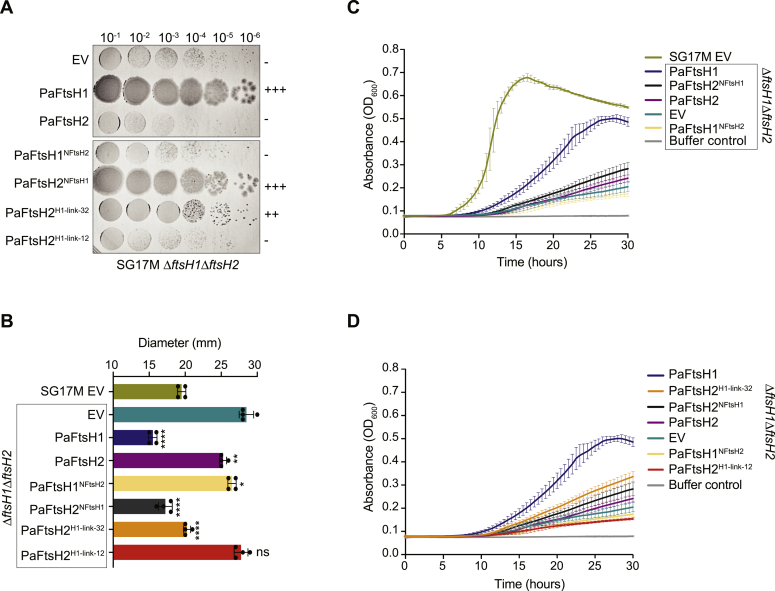
**MC**^**7+**^**linker identity determines the strength of complementation in *Pseudomonas aeruginosa* SG17M Δ*ftsH1*Δ*ftsH2* cells.***A*, colony growth of *P. aeruginosa* SG17M Δ*ftsH1*Δ*ftsH2* double deletion strains upon complementation with an empty expression vector control pJN105 (EV), PaFtsH1, PaFtsH2, PaFtsH1^NFtsH2^, PaFtsH2^NFtsH1^, PaFtsH2^H1-link-32^, or PaFtsH2^H1-link-12^ as indicated. Strains were grown overnight at 37 °C on LB plates with 30 μg/ml gentamicin. “+” and “-” scoring system relates the qualitative degree of colony growth of a particular strain genotype relative to that of SG17M EV (++++; wild-type (WT) colony growth) or SG17M Δ*ftsH1*Δ*ftsH2* (-; very poor colony growth). *B*, measurement of zone of inhibition upon exposure to the aminoglycoside antibiotic tobramycin. *P. aeruginosa* SG17M Δ*ftsH1*Δ*ftsH2* double deletion strains were complemented by expression with PaFtsH1 and PaFtsH2 WT and hybrid constructs as indicated. WT SG17M strain harboring an empty expression vector (EV) is shown for reference. A bacterial suspension of 0.5 Farland was distributed onto a Mueller Hinton agar plate containing 30 μg/ml gentamicin. The diameter of the zone of growth inhibition was measured after incubation at 37 °C for 20 to 24 h in the presence of a 10 μg tobramycin disk. ∗∗∗∗*p* < 0.00002; ∗∗∗*p* < 0.0002; ∗∗*p* < 0.002; ∗*p* < 0.02; ns: not significant (compared to “EV” (*teal* bar)) as analyzed by an unpaired *t* test. *C* and *D*, growth of *P. aeruginosa* SG17M WT or Δ*ftsH1*Δ*ftsH2* double deletion strains complemented by plasmid-borne expression of the indicated PaFtsH variants in M63-citrate minimal media. Identical growth curves for *P. aeruginosa* SG17M Δ*ftsH1*Δ*ftsH2* EV, complemented with PaFtsH1 and complemented with PaFtsH2 are displayed in (*C*) as reference values. EV, pJN105 empty expression vector control.

PaFtsH2^H1-link-32^ complementation of SG17M Δ*ftsH1*Δ*ftsH2* colony size was substantial, but less complete than PaFtsH2^NFtsH1^ complementation, and far greater than complementation by PaFtsH2 alone (Fig. 4*A*). PaFtsH2^H1-link-12^ did not rescue colony growth. We also monitored the ability of different FtsH linker chimeras to complement tobramycin susceptibility and growth in liquid M63-citrate minimal media (Figs. 4, *B*–*D* and S7). Expression of PaFtsH2^H1-link-32^, but not PaFtsH2^H1-link-12^, restored tobramycin tolerance to a level similar to that afforded by PaFtsH1 or PaFtsH2^NFtsH1^ (Fig. 4*B*). Similarly, while even complementation with PaFtsH1 was poor, only PaFtsH2^NFtsH1^ and PaFtsH2^H1-link-32^ partially complemented the slow-growth defect of SG17M Δ*ftsH1*Δ*ftsH2* in M63-citrate medium better than PaFtsH2 itself (Fig. 4, *C* and *D*). Thus, although *in vitro* and *in vivo* activities of hybrid proteins are not entirely congruent, as distinct substrate(s), adaptor proteins, or protein conformations might be determinative for recovery of *in vivo* phenotypes such as colony size, the PaFtsH1-PaFtsH2-linker hybrids significantly gained functionality in all or selected assays.

### Membrane-cytoplasmic linker across phylogeny

Previous phylogenetic analyses of representative homologs of PaFtsH1 and PaFtsH2 with highest sequence similarity in different taxonomic groups of bacteria revealed that similarity among PaFtsH1-like proteins was largely in line with overall phylogenetic relationships (as determined by 16S rRNA), whereas PaFtsH2-like protein similarity was not (5). These data are consistent with Pa*ftsH2*-like genes frequently horizontally transferred as part of the tLST genomic island into distinct genetic backgrounds of diverse bacterial species including the occasional pathogens *P*. *aeruginosa*, *Cronobacter sakazakii*, *Klebsiella pneumoniae*, and *E. coli* (5, 12, 13).

**Figure 5:**
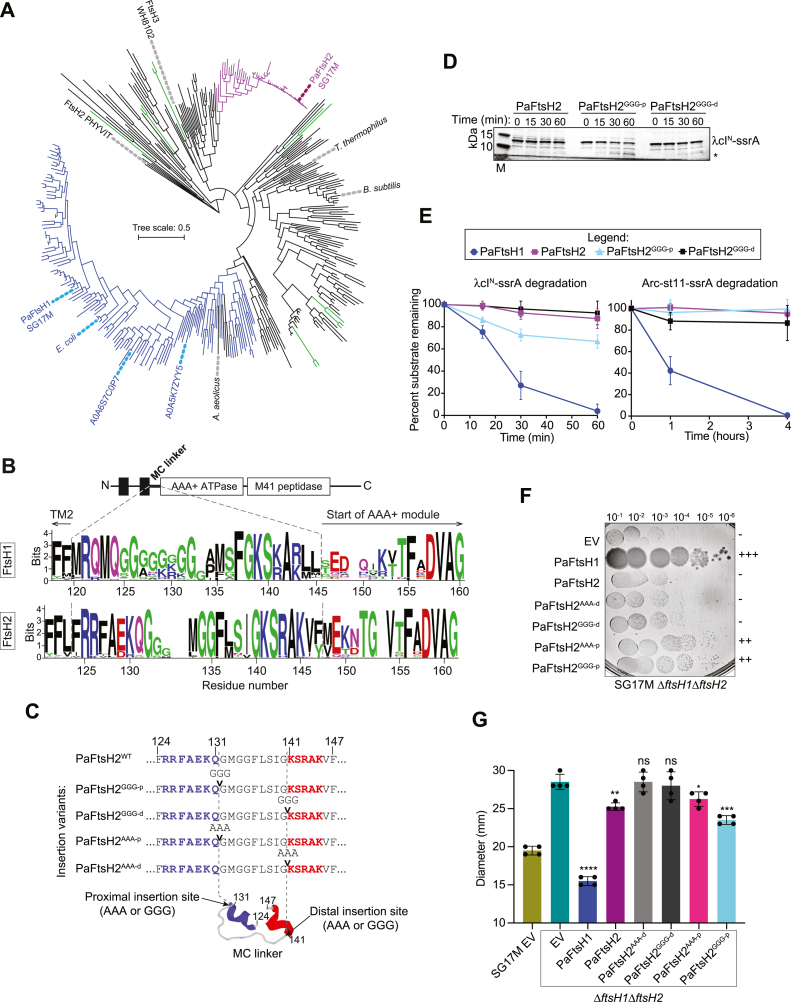
**Insertion of triplicate alanine or glycine residues in the membrane-proximal region of the PaFtsH2 MC linker activates PaFtsH2 *in vivo* and *in vitro*.***A*, evolutionary tree of full-length FtsH proteins of PaFtsH2 and PaFtsH2 homologs of >50% identity, representative FtsH proteins from major bacterial phyla and representative FtsH proteins from the archaeal and eukaryotic kingdoms (see Table S2). *Blue* branches indicate PaFtsH1-like proteins with a PaFtsH1-like linker, and *red* branches indicate PaFtsH2 proteins with a PaFtsH2-like linker. *Green* branches indicate *Arabidopsis thaliana* FtsH proteins. Tree scale indicates amino acid substitutions per site. Sequence alignment was performed with ClustalX2 and manually curated. The phylogenetic tree was constructed using the maximum likelihood of distance estimation with MEGA 7.0 using standard parameters with 100 bootstrap iterations. *B*, domain structure of a model FtsH enzyme and WebLogos of typical FtsH1 and FtsH2 MC^7+^ linker sequences constructed from 149 sequences of PaFtsH1-like proteases (*blue* branches in (*A*)) and 48 sequences of PaFtsH2-like (*red* branches in (*A*)). Extended WebLogo depicts the MC^7+^ linker region. Residue numbers refer to PaFtsH1 and PaFtsH2, respectively. *C*, a zoom-in on the MC linker sequences of indicated PaFtsH2 triplicate residue insertion variants. PaFtsH2 MC linker structural prediction was generated by PEP-FOLD 3.5 (bottom; (71)) where *blue* and *red* helices indicate predicted proximal and distal helices, respectively, and the course grained energy (sOPEP) is −35.74. Site of proximal insertion is between amino acids 131 and 132, and site of distal insertion is between amino acids 140 and 141. *Arrows* indicate positions of residue insertions (Gly-Gly-Gly or Ala-Ala-Ala) for four PaFtsH2 insertion variants: PaFtsH2^GGG-p^, PaFtsH2^GGG-d^, PaFtsH2^AAA-p^, PaFtsH2^AAA-d^. *D*, degradation of λcI^N^-ssrA (15 μM) was monitored in the presence of PaFtsH2, PaFtsH2^GGG-p^, or PaFtsH2^GGG-d^ (3.04 μM hexamer equivalents) as described in Figures 1*E* and 3*C*. Bands corresponding to full-length λcI^N^-ssrA and a ∼5.7 kDa degradation intermediate product (denoted by *asterisk*) are indicated. M: Molecular weight marker. *E*, quantification of full-length substrate (λcI^N^-ssrA and Arc-st11-ssrA) remaining over the course of the reaction time. Error bars represent SD of experiments performed in triplicate. Data for degradation reactions containing PaFtsH1 and PaFtsH2 are the same as in Figure 3*G*. *F*, colony growth of *P. aeruginosa* SG17M Δ*ftsH1*Δ*ftsH2* double deletion strains complemented by the expression of the indicated PaFtsH variants. “+” and “-” scoring system relates the qualitative degree of colony growth of a particular strain genotype relative to that of SG17M EV (pJN105) (++++; wild type colony growth) or SG17M Δ*ftsH1*Δ*ftsH2* EV (-; very poor colony growth). *G*, measurement of zone of inhibition upon treatment with the aminoglycoside antibiotic tobramycin. *P. aeruginosa* SG17M Δ*ftsH1*Δ*ftsH2* double deletion strains were complemented by expression with PaFtsH1 and PaFtsH2 wild-type and triplicate insertion constructs as indicated. Wild-type SG17M strain harboring an empty pJN105 expression vector (EV) is shown for reference. ∗∗∗∗*p* < 0.00002; ∗∗∗*p* < 0.0002; ∗∗*p* < 0.002; ∗*p* < 0.02; ns: not significant (compared to “EV” (*dark teal* bar)) as analyzed by an unpaired *t* test.

Given our structure/function data on the impact of the MC^7+^ linker in PaFtsH1 *versus* PaFtsH2 activity, we hypothesized that MC^7+^ linker similarity is congruent with the clustering of FtsH proteins. To this end, we aligned representative FtsH proteins from different genera of γ-proteobacteria from the NCBI and UniProt databases homologous to PaFtsH1 and PaFtsH2 and also included FtsH representatives from all established major phyla of the bacterial phylogenetic tree. As FtsH proteins are also highly conserved in other major evolutionary branches of life including amoeba, fungi, plants, and mammals, FtsH representatives from select organisms, such as the model plant *Arabidopsis thaliana* (which possess 11 FtsH homologs) and *Homo sapiens*, were also added to this analysis. We generated a protein evolutionary tree where branch length represents the number of sequence changes between entries (Fig. 5*A*; (5)). As expected, the PaFtsH1 and PaFtsH2 representatives each clustered together, but far apart from each other. PaFtsH1-like proteins (blue branches; >46% identity) and PaFtsH2-like proteins (purple branches; >61% identity) were therefore used to generate two sequence alignments, to allow comparison between the MC linkers of the representative proteins which originated from different bacterial phyla (Table S2 and Fig. 5*B*).

Comparing the amino acid preferences within the MC^7+^ linker sequences (including the first seven residues of the AAA+ modules) shows a strikingly conserved glycine-rich character of the PaFtsH1-type linker in contrast to the PaFtsH2-type linker (Fig. 5*B*). Whereas up to eight clustered glycines (seven being common, *e.g., GGGGG[K/R]GG*) occurred in PaFtsH1-type linkers, those of the PaFtsH2-like proteases contained fewer glycines and no long Gly-rich blocks (*e.g. GMGG* being common). In addition, the Gly-rich sequence tracks in both types of linkers are positioned differently with respect to the TM2 transmembrane helix, which is extended in length in PaFtsH2, and the AAA+ module (Fig. 5*B*).

Subsequently, we dissected the more immediate linker evolution in more detail. Besides for one protein, the PaFtsH2 linker has been conserved in all 287 full-length FtsH2 homologs with an identity >80% as retrieved from the NCBI and UniProt databases (Fig. S8*A*). This finding indicates that, despite horizontal gene transfer into different species, the FtsH2 linker sequence had remained stable. Few more diverged (longer, more glycine-rich) linkers are observed among 252 FtsH2 homologs with an identity larger than 67% (UniProt database), suggesting that immediate, but not accelerated, linker evolution of similar proteins can occur in different species (Fig. S8*B*). Equally, only five out of 252 full length PaFtsH1 proteins of *P. aeruginosa* with identity >50% and >90% query coverage (BLAST database) showed evolved linkers (Fig. S8*C*). All five evolved linkers displayed reduced glycine content suggesting that longer more glycine rich linkers are not evolutionary supported in *P. aeruginosa*. In 248 non-*P. aeruginosa* representative homologs of PaFtsH1 with identity >74.7% (UniProt database), few linkers with extended length and glycine content have been observed, besides one reduced linker sequence (Fig. S8*D*). However, the FtsH linker has been subject to evolution already in isolates from deeply branching phyla such as Bipolaricaulota, Caldiserica, Dictyoglomota, and Coprothermobacterota (Fig. S9). Although most isolates display FtsH proteins with short glycine-poor linker sequences, longer glycine-rich linkers have occasionally evolved.

### Insertion of Gly-Gly-Gly membrane-proximal into the MC linker activates PaFtsH2

The MC linker in PaFtsH2^H1-link-12^ contains more glycines than that of wild-type (WT) PaFtsH2, is longer by one residue, and activates proteolysis of λcI^N^-ssrA (Fig. 3, *A*, *F*, and *G*). To probe linker length, flexibility and positional effects of a 3x-glycine stretch within the linker, we cloned, overexpressed and purified (with the exception of PaFtsH2^AAA-p^), PaFtsH2 variants with three glycines or alanines inserted immediately membrane-proximal (PaFtsH2^GGG-p^ and PaFtsH2^AAA-p^) or membrane-distal (PaFtsH2^GGG-d^ and PaFtsH2^AAA-d^) to the WT MC linker. These insertions are C-terminal of Gln131 and Gly140, respectively, and thereby directly at the sequence junctions between predicted structured and unstructured regions of the PaFtsH2 MC linker peptide fold corresponding also to PaFtsH1 (Fig. 5*C*). PaFtsH2^GGG-p^, but not PaFtsH2^GGG-d^ partially degraded λcI^N^-ssrA over the course of 1 h (Fig. 5, *D* and *E*). Consistent with this, plasmid-borne expression of both PaFtsH2^GGG-p^ and PaFtsH2^AAA-p^ partially rescued the colony-size defect of SG17M Δ*ftsH1*Δ*ftsH2*, albeit less well than PaFtsH1, but better than the PaFtsH2 parent (Fig. 5*F*). Of note, PaFtsH2^GGG-p^ performed better than PaFtsH2^AAA-p^ in the tobramycin assay (Fig. 5*G*). Neither PaFtsH2^GGG-d^ nor PaFtsH2^AAA-d^ rescued colony growth (Fig. 5*F*), scored well in the tobramycin assay (Figs. 5*G* and S7), or complemented growth in M63-citrate liquid medium (Fig. S10*A*). However, both linker variants had reduced rates of ATP hydrolysis (as measured at 45 °C, the temperature optimum for PaFtsH1) which might partially account for their poor activities (Fig. S10*B*). In combination, these results strongly suggest that MC linker length and flexibility influence the biological activity of FtsH proteases in ways that depend on the position of a flexible segment within the linker.

**Figure 6:**
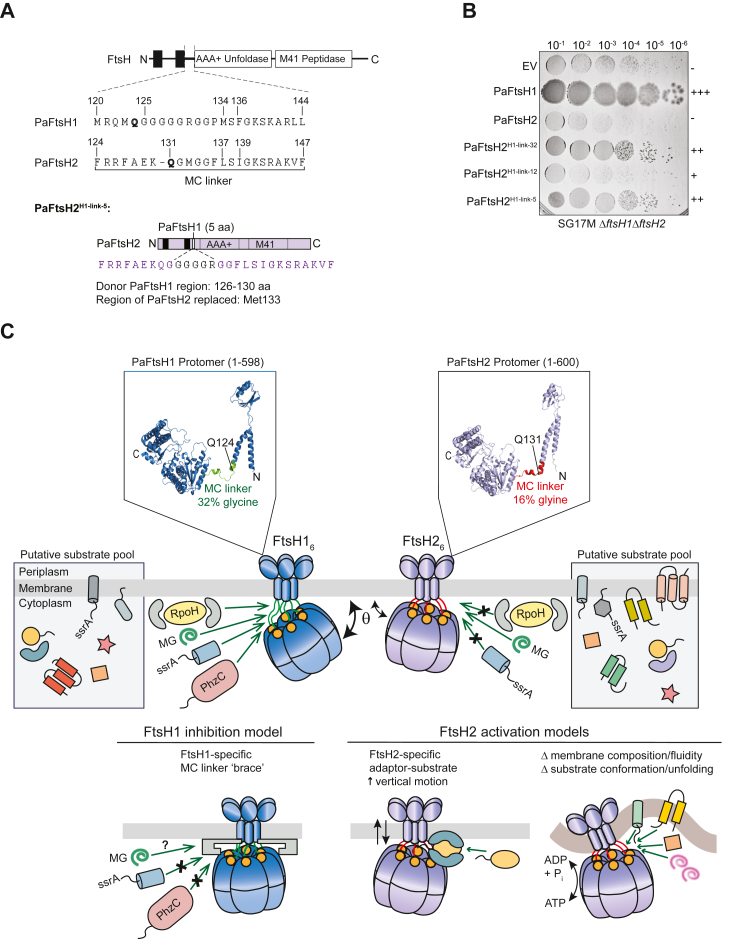
**Membrane-cytoplasmic linker properties are important in determining PaFtsH1 and PaFtsH2 functionality.***A*, schematic description of the PaFtsH2^H1-link-5^ variant. Met^133^ of PaFtsH2 was replaced with a stretch of five amino acids from PaFtsH1, GGGGR (residues 126–130). PaFtsH1 Gln^124^ and PaFtsH2 Gln^131^ are denoted in *bold* and are predicted to localize at the base of TM2 in both enzymes, as referred to in *panel C* (*top*). Amino acids labeled in *purple* on the PaFtsH2^H1-link-5^ variant schematic are of PaFtsH2 identity (*bottom*). *B*, colony growth of *Pseudomonas aeruginosa* SG17M Δ*ftsH1*Δ*ftsH2* double deletion strains complemented with either empty vector pJN105 (EV), wild-type Pa*ftsH1*, Pa*ftsH2*, or the hybrid constructs PaFtsH2^H1-link-32^, PaFtsH2^H1-link-12^, and PaFtsH2^H1-link-5^, as indicated. The plate in this panel is the same as in Figure 4*A* for EV, PaFtsH1, and PaFtsH2. “+” and “-” scoring system relates the qualitative degree of colony growth of a particular strain genotype relative to that of SG17M EV (++++; wild-type colony growth) or SG17M Δ*ftsH1*Δ*ftsH2* (-; very poor colony growth). *C*, model of MC linker–dependent functionality of FtsH proteases. The more flexible MC linker of PaFtsH1 compared to that of PaFtsH2 (32% and 16% glycine, respectively) allows enhanced motion relative to the inner membrane plane (θ) which may promote access of substrates into the FtsH1 axial pore (highly conserved FVG pore loops are indicated by *orange-filled circles*). The comparatively reduced MC linker flexibility of PaFtsH2 restricts its plane of motion and inhibits the productive entry of substrates for proteolysis by PaFtsH2. *Green* arrows indicate productive substrate recognition and subsequent proteolysis and arrows with a black “X” indicate unproductive substrate recognition. PaFtsH2 degrades molten-globule-like (MG) substrates *in vitro*, which may indicate an *in vivo* prerequisite for the remodeling of folded substrates prior to PaFtsH2-dependent degradation. Alternatively, the higher ATPase activity/substrate pulling force of PaFtsH2 may point to a scenario in which PaFtsH2 preferentially proteolyzes integral membrane proteins compared to PaFtsH1, a substrate class that we did not test *in vitro*. As Pa*ftsH2* is found on a tLST genomic island which confers enhanced tolerance to heat and other stresses to the host organism, it is possible that a higher membrane fluidity characteristic of higher temperatures is required for the optimal function of PaFtsH2 or that condition-dependent adaptors or factors are required for the specific delivery of substrates. Although PaFtsH2 has a more rigid MC linker than PaFtsH1 and its horizontal movement relative to the membrane plane is constrained, it contains a longer TM2 domain which may result in higher vertical ‘up-and-down’ movement within the inner membrane. This vertical movement may be important for the degradation of certain substrates or for regulating substrate entry into the PaFtsH2 axial pore. As PaFtsH1 was active against most protein substrates tested in our study, models for its inhibition *in vivo* include those that restrict or “brace” the flexibility of its MC linker such that entry into its axial pore is no longer accessible. Monomer structural predictions of PaFtsH1 (*top left*, Q9HV48_PSEAE) and PaFtsH2 (*top right*, A0485GIM3_PSEAI) were generated using AlphaFold. Although the structures are torsionally inaccurate, they are helpful for viewing the proximity of the MC linkers to the transmembrane (towards the N terminus) and cytoplasmic (towards the C terminus) domains. Conserved Gln (Q) residues as mentioned in panel A are located at the base (C-terminal end) of TM2 of each protein and labeled with arrows. The UniProt ID used for modeling PaFtsH2 is 99.52% identical to the PaFtsH2 sequence from SG17M clone C. The three residues that differed from PaFtsH2 (S127, M447, E524) were changed to residues of PaFtsH2 identity using PyMol (S127R, M447I, E524A). Only residues 1-598 of PaFtsH1 and 1-600 of PaFtsH2 are shown in the figure, as the C-terminal regions are predicted to be highly unstructured.

The fact that PaFtsH2^GGG-p^ has biological activities that PaFtsH2 lacks suggests that the WT MC linker of PaFtsH2 lacks flexibility and length (Fig. 6), but this model fails to explain why PaFtsH2^H1-link-12^ was even less active than PaFtsH2^GGG-p^ in some *in vivo* assays. We noticed, however, that a glutamine at position 131 (Gln^131^) in PaFtsH2 near the membrane-proximal end of the MC^7+^ linker (Gln^124^ in PaFtsH1), is highly conserved in PaFtsH2 sequences and the vast majority of PaFtsH1 linkers (Figs. 5*B*, S1, and S7). As the glutamine at this position is absent in the construct PaFtsH2^H1-link-12^ inactive *in vivo* (Fig. 3*B*), we constructed an additional PaFtsH2 chimera that preserves Gln^131^ and adds the glycine-rich region of five residues from PaFtsH1 at the respective aligned position, basically replacing Met^133^ (PaFtsH2^H1-link-5^; Fig. 6*A*). Notably, PaFtsH2^H1-link-5^ expression rescued the colony growth defect of SG17M Δ*ftsH1*Δ*ftsH2* to a similar extent as PaFtsH2^H1-link-32^ (which also retains Gln^131^) (Fig. 6*B*). Combined structural and topological predictions from AlphaFold models and transmembrane helix prediction programs place Gln^131^ at the base of the TM2 helix that ends C-terminally with FRRFAEK^130^ (a sequence preserved in all PaFtsH2 homologs; Figs. 5*B* and 6*C*). As Gln^131^ is localized to the TM2 C-terminal end, it is possible that this amino acid serves a functional role such as in aiding the formation of a structural boundary between TM2 and the MC linker, thereby influencing the flexibility of the membrane tether and *in vivo* activity.

## Discussion

Bacterial species occupy defined ecological habitats based on their physiological and metabolic properties and ability to tolerate different environmental stresses. *P. aeruginosa* is not only a successful environmental species (42), but an important opportunistic human pathogen that infects immune-compromised individuals and contributes substantially to the morbidly and mortality of cystic fibrosis patients (43). Of note, *P. aeruginosa* has been recently found to be one of the most frequently co-infecting pathogen in COVID-19 patients contributing to disease severity (44, 45). Compared to other strain clusters, clone C of *P. aeruginosa* is especially robust and abundant in the environment and causes acute and chronic human infections. Elucidating the molecular mechanisms that contribute to the prevalence of *P. aeruginosa* clones, groups of closely related strains with distinct habitat preferences, is therefore an important goal with both ecological and clinical impact. We have recently shown that *P. aeruginosa* clone C harbors the tLST genomic island, which includes Pa*ftsH2* and provides increased tolerance against elevated temperature and other stresses. It is therefore plausible that expression of tLST-encoded factors may contribute to establishing the observed abundant representation of *P. aeruginosa* clone C (11, 13, 42, 46, 47, 48). The presence of the Pa*ftsH2* xenolog in addition to core genome Pa*ftsH1* distinguishes *P. aeruginosa* clone C and clone J from the model *P. aeruginosa* strains PAO and PA14 and most other investigated model bacterial species, which encode only a single membrane-bound FtsH enzyme. Evolutionary analysis reveals that a very similar tLST genomic island containing an *ftsH2* gene has been transferred into and maintained in other bacterial species including in clones of occasional pathogens (13). On the other hand, in the same tLST context, *ftsH2* has also been observed to be frequently deleted indicating evolutionary constraints to maintain two *ftsH* genes in some genetic backgrounds. In general, however, expansion of FtsH proteases in bacteria is not as uncommon. For example, although *Burkholderia* species harbor a FtsH1 homolog that is closely related to PaFtsH1, some organisms have acquired additional distinct, distantly-related FtsH proteases (Fig. S11). It is well known that cyanobacteria harbor up to four FtsH paralogs and highly diverse xenologs which cluster with FtsH from other bacterial species (Fig. S11). Furthermore, phytoplasma, such as the vineyard-grape pathogen *Flavescence doree*, carry multiple virulence-associated and clearly paralogous *ftsH* genes despite having minimalized genomes (Fig. S11) (49, 50). Thus, expansion of FtsH protease variants appears to be an adaptive evolutionary strategy that has occurred independently in more than one bacterial species/sub-species throughout the phylogenetic tree and in higher organisms including plants and humans (Figs. S11 and 5*A* with reference FtsH proteases indicated). The phylogenetic relationships among the FtsH proteases suggest various mechanisms such as gene duplication with subsequent diversification and horizontal gene transfer to contribute to the multiplication of *ftsH* protease genes in genomes.

Building upon a previous study (5), here we characterized the molecular basis of functional differences between the PaFtsH1 and PaFtsH2 proteases of *P. aeruginosa* clone C*.* Notably, although both enzymes are active ATP-dependent proteases, we find that they have common and distinct substrate specificities as concluded from *in vivo* and *in vitro* studies ((5) and this work). For example, both enzymes degrade the structurally-relaxed protein β-casein, whereas only PaFtsH1 degrades λcI^N^-ssrA and Arc-st11-ssrA. We also demonstrate that many of the biochemical and biological activities of these PaFtsH xenologs depend on the identity and character of a short region of the linker that connects the TM2 transmembrane helix to the cytoplasmic AAA+ module. While tilting of the catalytic domains against the membrane plane provides substrate access (3), we show in this work that the impact of highly divergent MC linker sequences extends beyond a solely physical role in providing substrate access to a much more determinative and flexible role in substrate processing. Future work will identify natural substrates processed by FtsH proteases with distinct linker sequences.

### MC linker flexibility and control of FtsH function

We initially discovered that a hybrid protein containing the TM1 helix, periplasmic domain, TM2 helix, and MC^7+^ linker region of PaFtsH1 and the AAA+ module and protease domain of PaFtsH2 had cellular activities similar to PaFtsH1 and *vice versa.* Further sequence swaps identified a short sequence stretch of the cytoplasmic MC^7+^ linker as being substantially responsible for functionality of the PaFtsH2 catalytic subunits such as PaFtsH1-like substrate degradation *in vitro* and phenotypic recovery *in vivo*. Indeed, swapping as few as five residues from the PaFtsH1 MC linker into PaFtsH2 was sufficient to endow the chimeric protease with substantial PaFtsH1-like activities (Fig. 6, *A* and *B*).

In the hexameric FtsH enzyme, the MC^7+^ linker appears to determine the flexibility of the cytoplasmic parts of the protease relative to the membrane (Fig. 6*C*). This model is consistent with (i) predictions of the flexibilities of different linkers that reinforce or fail to support certain PaFtsH1-specific functions; (ii) with the higher glycine content of the MC linker of PaFtsH1 relative to PaFtsH2; (iii) with low-resolution EM structures of PaFtsH1, PaFtsH2, and an active chimeric enzyme; and (iv) with the observation that position-specific insertion of just three glycines or alanines into the MC linker of PaFtsH2 allowed this chimera to partially restore defects caused by an *ftsH1* deletion.

Our results advocate the hypothesis that higher MC linker flexibility correlates with PaFtsH1-like biochemical and biological functions. Identification of endogenous biological substrates of *P. aeruginosa* PaFtsH2, however, remains to be determined, as does addressing the question of how the properties of the PaFtsH2 MC linker contribute to its proteolytic specificity. Intracellular levels of PaFtsH2 increase during nutrient deprivation and it represents the predominant FtsH species in late-stationary phase (5). Thus, PaFtsH2-specific substrates, which may include the gene products of the tLST genomic island (46, 51), may be more prevalent in late-stationary phase or during biofilm formation, whereby differences in substrate specificity may provide a survival benefit to the organism. For example, the faster rate of ATP hydrolysis of PaFtsH2 compared to PaFtsH1 may aid in the processing of thermodynamically more stable substrates. ATP-hydrolysis activity by PaFtsH2 also has a temperature optimum that is higher than that of the PaFtsH1 enzyme (Fig. S2*B*). This fact indicates superior PaFtsH2 functionality at higher temperatures consistent with glycine-poor linker sequences of FtsH proteases in thermophilic organisms such as *Thermus* spp. and even FtsH1-like *A. aeolicus* (Fig. S1). *P. aeruginosa* skin infections in humans are a frequent issue associated with hot tub usage and outbreaks on thermally sterilized endoscopes by sequence type ST17 (clone C) have occurred (52, 53); these high-temperature environments may favor and/or activate PaFtsH2 due to its more rapid ATPase rate. Alternatively, increased membrane fluidity or change in membrane composition could suppress the apparent ‘negative gating’ properties of PaFtsH2's ‘rigid’ MC linker and thus contribute to survival of the organism (Fig. 6*C*).

Moreover, the presence of protein adaptors able to alter PaFtsH1 or PaFtsH2 activity and function (*e.g.*, HflC and HflK) may also change depending upon specific growth or environmental conditions. An interesting and testable hypothesis is that some adaptors/regulators exert their influence on FtsH activity, at least in part, by abrogating or mimicking the effects of a flexible *versus* stiff attachment to the inner membrane. Surprisingly, such physiological impact seems to have developed gradually as selectively investigated deeply branching bacterial phyla contain predominantly, but not exclusively, FtsH proteins with short linkers (Fig. S8*A*). Like the MC linker of *P. aeruginosa* PaFtsH2, MC linkers from well-investigated extremophile variants of FtsH (*e.g.*, *A. aeolicus* and *Thermus thermophilus*) or single-membrane Gram-positive bacteria (*e.g.*, *M. tuberculosis*) also have fewer glycines (Fig. S1). This observation emphasizes the need to elucidate molecular mechanisms that determine how MC linker flexibility and/or properties impact FtsH function and overall cellular physiology (54). Our work demonstrates the functional importance of PaFtsH MC^7+^ linkers with implications that potentially extend to other membrane-tethered FtsH proteases and even membrane proteins in all kingdoms of life. Although linkers have previously been identified as mediating signal transduction (55, 56, 57, 58), we have shown that the molecular properties of the junction separating transmembrane and cytoplasmic domains of a protein can have profound significance on its activity and physiological impact. These findings anticipate further that molecular hinges found in other membrane-bound proteins may be key regulatory elements to a greater extent than previously anticipated.

## Experimental procedures

### Strains and plasmids

*P. aeruginosa* strains used in this study were the *P. aeruginosa* clone C strain SG17M (42) and its derivative SG17M053 (SG17M Δ*ftsH1*Δ*ftsH2*) (5). For liquid growth, strains were cultivated in LB broth medium (BD Difco) at 37 °C. If needed, 30 μg/ml gentamicin (for pJN105 plasmid selection in *P. aeruginosa* and *E. coli*) and 50 μg/ml kanamycin (for pET-28a(+) (Novagen) expression plasmid selection in *E. coli*) were used. *P. aeruginosa* SG17M genomic DNA was used as a template to amplify native Pa*ftsH1* and Pa*ftsH2* sequences. To generate constructs for protein overexpression, standard cloning of the open reading frames with upstream Shine-Dalgarno sequences and downstream codons for a 6xHis-tag into the expression vector pET-28a(+) between the NheI/XbaI restriction sites was performed. For cloning and expression of WT and variant Pa*ftsH1* and Pa*ftsH2* genes in *P. aeruginosa* SG17M, the L-arabinose inducible broad host-range expression vector pJN105 was used (5, 59) combined with the Pa*ftsH2* Shine-Dalgarno sequence to normalize protein production. For the construction of Pa*ftsH* DNTR hybrid constructs (PaFtsH1^NFtsH2^, PaFtsH2^NFtsH1^ and constructs a–e), the desired regions of Pa*ftsH1* and Pa*ftsH2* were selectively amplified from *P. aeruginosa* SG17M genomic DNA, isolated, subjected to overlapping PCR to create custom hybrids, and cloned into pJN105. Standard Gibson Assembly (New England Biolabs) cloning was used to construct linker variants PaFtsH2^H1-link-32^, PaFtsH2^H1-link-12^, and PaFtsH2^H1-link-10^; and site-directed mutagenesis (QuickChange, Agilent) was used to create all triplicate insertion MC linker variants in PaFtsH2. Some constructs were subcloned into pJN105, while others were directly cloned and constructed in pJN105. All constructs were confirmed by Sanger sequencing. Plasmids used in this study are described in Table S3.

All primer sequences used in this study are described in Table S4. Stable introduction of expression plasmids into *P. aeruginosa* SG17M Δ*ftsH1*Δ*ftsH2* was achieved by electroporation using Gene Pulser (Bio-Rad) with 0.1 cm gap cuvettes operated at 13 kV/cm, 400 Ω and 25 μF.

### Protein expression and purification

For protein induction, EcFtsH-Myc-6xHis, PaFtsH1-6xHis, and PaFtsH2-6xHis variants were expressed from a pET-28a(+) plasmid in *E. coli* strain BL21(DE3) (New England Biolabs) grown at 37 °C in LB supplemented with 50 μg/ml kanamycin. At OD_600_ of 0.6, temperature was reduced to 30 °C, and β-D-1-thiogalactopyranoside was added to a final concentration of 0.5 mM in 1 L cultures and cells were harvested after 3 h. FtsH purification was performed as previously described with modifications (24, 60). Cell pellets were resuspended in Tris Buffered Saline containing protease inhibitor (Pierce Protease Inhibitor Capsules, EDTA-free, Thermo Fisher Scientific) and frozen. For protein purification, cell suspensions were pelleted by centrifugation and pellets were lysed by repeated freeze thaw cycles (4×) in which frozen cell pellets were thawed in a warm water bath and frozen in a dry ice and ethanol bath. The lysate was mixed at room temperature with 20 ml B-PER (a mild non-ionic detergent; Thermo Fisher Scientific) containing 10 mg lysozyme, 5 μl benzonase (Merck), and EDTA-free protease inhibitor for 20 min. Lysate was then centrifuged for 30 min at 30,000 RCF at 4 °C, and supernatant was discarded. The resulting pellet containing inclusion bodies was resuspended in solubilization buffer (50 mM N-cyclohexyl-3-aminopropanesulfonic acid (pH 11.0), 2 mM MgSO_4_, 0.1 mM ZnCl_2_). After manual resuspension, ATP was added to a final concentration of 1 mM, followed by a dropwise addition of 15% *N*-lauroylsarcosine (Sigma) to a final concentration of 0.6%. The solution was mixed at room temperature for 30 min to allow for the solubilization of inclusion bodies and then centrifuged for 30 min at 30,000 RCF. The supernatant was applied to a Ni^2+^ Sepharose column (5 ml HisTrap HP; Cytiva) equilibrated in base buffer (50 mM Tris-HCl (pH 8.0), 100 mM NaCl, 20 mM imidazole, 5 mM MgSO_4_, 2 mM β-mercaptoethanol, 100 μM ZnCl_2_, 1 mM ATP) containing 0.6% *N*-lauroylsarcosine. The column was washed with 3 column volumes (CV) of the same buffer, and then subjected to a wash with 9 CV of buffer made of base buffer plus 0.5% NP-40 (note that *N*-lauroylsarcosine is omitted from this wash buffer to allow for protein refolding). The column was washed with 3 CV by a third and final solution composed of base buffer plus 10% glycerol, 0.1% NP-40, and 80 mM imidazole (the first step of a stepwise elution; we find FtsH is still bound to the column at this step). Finally, protein was eluted with 280 mM imidazole in base buffer containing 10% glycerol, and 0.1% NP-40. Fractions containing protein at >95% purity as judged by an 4 to 20% gradient SDS-PAGE gel (Mini-PROTEAN TGX Precast, Bio-Rad) stained with Coomassie brilliant blue R-250 (BioRad) were combined, concentrated, and dialyzed overnight at 4 °C in dialysis buffer (50 mM Tris-HCl (pH 8.0), 80 mM NaCl, 60 mM imidazole, 5 mM MgSO_4_, 2 mM β-mercaptoethanol, 10 μM ZnCl_2_, 10% glycerol). Precision Plus Protein Dual Color Standard (Bio-Rad) was consistently used as molecular weight marker. We ruled out the possibility of contaminating host *E. coli* FtsH associating with PaFtsH complexes after analyzing the migration patterns of purified PaFtsH1 and PaFtsH2 on 4 to 20% SDS-PAGE gels, as EcFtsH, PaFtsH1, and PaFtsH2 possess different sizes (monomer molecular weights: EcFtsH-Myc-6xHis: 73,127 Da; PaFtsH1-6xHis: 70,886 Da; PaFtsH2-6xHis: 69,521 Da) and run distinctly on the SDS-PAGE gels (Fig. 1, *F* and *G*). After dialysis, purified protein was concentrated, aliquoted, flash frozen in liquid nitrogen, and stored at −80 °C.

For purification of radioactive proteins (^35^S-EcFtsH, ^35^S-PaFtsH1 and ^35^S-PaFtsH2), the same experimental procedure as above was followed with the following modifications to the protein induction conditions: *E. coli* BL21(DE3) (New England Biolabs) cells harboring a pET-28a(+) expression plasmid encoding FtsH-6xHis were grown in 500 ml of M9 minimal media lacking methionine and cysteine at 37 °C and induced at OD_600_ 0.6 with 0.5 mM β-D-1-thiogalactopyranoside. At the time of induction, 20 μCi/ml EasyTag Express ^35^S Protein Labeling Mix (PerkinElmer) was added to cultures and temperature was reduced to 30 °C. After 3 hours of growth post-induction, cells were harvested *via* centrifugation and purification was carried out as described above.

For determination of the native molecular weights of purified FtsH enzymes, calibration curves were calculated by running molecular weight standards (BioRad cat. No. 151-1901) on a Superdex increase 3.2/3.0 analytical gel filtration column equilibrated in Buffer PD (50 mM Tris-HCl (pH 8.0), 80 mM NaCl, 5 μM MgSO_4_, 12.5 μM ZnCl_2_, 10% glycerol, 2 mM β-mercaptoethanol, and 0.1% NP-40). Purified FtsH proteins were applied to the column and native molecular weights were calculated by fitting to a one-phase decay model.

Purification of the ssrA tagged substrates, the P22 Arc repressor with the (6 x H)KNQHE (st11) tag (Arc-st11) Arc-st11-ssrA and the N-terminal domain of the phage λ repressor cI, λcI^N^, substrates were carried out as described (61, 62).

### Growth assays

For assessing growth on solid media, WT *P. aeruginosa* SG17M and SG17M Δ*ftsH1*Δ*ftsH2* mutant harboring pJN105 empty vector control and derivatives with cloned PaFtsH WT and variants were inoculated from a single colony into 10 ml LB medium overnight supplemented with 30 μg/ml gentamicin. The OD_600_ of the cell suspension was adjusted to 1 in LB medium and a 10-fold serial dilution was prepared in a 96-well plate. Ten microliters of each suspension was spotted onto an LB-agar plate with 30 μg/ml gentamicin and incubated at 37 °C overnight. Colony size as a semiquantitative assessment of growth was monitored visually and imaged at different time points as described previously (5). For quantifying growth in liquid culture, bacteria were grown overnight in LB and resuspended to a final OD_600_ of 1.0 in PBS. One microliter of suspension was added to 200 μl M63 minimal medium with the appropriate antibiotics in 96-well plates. Growth conditions were provided in a SpectraMax i3x (Molecular Devices), where the plate was incubated at 37 °C under shaking for 24 h with OD_600_ measured every 30 min.

### Antibiotic susceptibility assays

*P. aeruginosa* SG17M and/or respective derivatives grown on LB-agar plates overnight were resuspended in PBS to a final OD_600_ of 0.1. An aliquot of this suspension was streaked out evenly on a Mueller Hinton agar plate (containing 30 μg/ml gentamicin) to which two tobramycin discs (10 μg, Liofilchem) was subsequently placed central on the agar plate. The tobramycin-disc-containing plate was incubated at 37 °C and the diameter of the inhibition zone was measured after 20 to 24 h.

### Enzymatic assays

Protein degradation assays were performed at 40 °C in Buffer PD supplemented with 5 mM ATP and an ATP regeneration system consisting of 50 μg/ml creatine kinase (Millipore-Sigma) and 5 mM creatine phosphate (Millipore-Sigma). Protein substrates were added to degradation reactions at the following concentrations: 40 mM β-casein (Sigma), 15 μM Arc-st11-ssrA, 15 μM λcI^N^-ssrA, and 15 μM untagged λcI^N^, respectively. For Arc-st11-ssrA degradation reaction, PaFtsH1/2 was added at a final concentration of 3.53 μM hexamer equivalents, and for reactions containing all other protein substrates, 3.04 μM PaFtsH1/2 hexamer equivalents were added. Reactions were quenched by the addition of SDS-loading buffer and boiled before separation by a 4 to 20% SDS-PAGE (BioRad). Proteins in gels were stained with SYPRO orange (Sigma-Aldrich), and gels were imaged with a Typhoon FLA9500 scanner (GE Healthcare) and quantified with ImageQuant 8.1 software (GE Healthcare). The fraction of substrate remaining at each reaction time point was calculated by dividing the substrate band intensity by the substrate band intensity at time zero.

ATP hydrolysis assays were performed at 40 °C using an NADH-coupled assay (63) containing 0.43 μM FtsH hexamer, 5 mM ATP, and NADH-coupled regeneration system in Buffer PD. ATP hydrolysis was assessed by the decrease in absorbance at 340 nm using a SpectraMax M5 plate reader (Molecular Devices). In all ATP hydrolysis measurements, the background rate of ATP depletion in the absence of protease was subtracted from all ATPase reactions for calculation of protease-dependent ATPase rates.

### Peptide arrays

Arrays of 12-mer peptides were synthesized by standard Fmoc (9-fluorenylmethoxycarbonyl) solid-phase techniques and C-terminally linked to a cellulose membrane using a ResPep SL peptide synthesizer (Intavis). Each peptide spot on the arrays contained a sliding window of 12 amino acids with a step size of two amino acids starting from the N-terminus and moving towards the C-terminus corresponding to the sequences of target proteins indicated on the respective figures and in the figure legends (PaRpoH, PaGlmM, PaLepB1, PaMinD, PaPhzC1/2 (identical)). Each peptide array was probed with 1 μM ^35^S-EcFtsH, ^35^S-PaFtsH1, or ^35^S-PaFtsH2 in the presence of 1.25 mM ATPγS. Peptide sequences corresponding to each spot are listed in Table S1. Arrays were incubated with gentle agitation in TBST (3 x 5 min) followed by overnight blocking in 5% BSA in TBST at 4 °C. Blocked arrays were washed in TBST (2 × 5 min) followed by Buffer PD containing 0.03% NP-40 (2 × 5 min) at room temperature, and then with fresh Buffer PD containing 0.03% NP-40, 1.25 mM ATPγS, 0.05% BSA, and 1 μM radioactive FtsH hexamer. After incubation with agitation for 2 h at room temperature, the array was briefly washed with Buffer PD containing 0.03% NP-40 and 1 mM ATPγS (2 × 30 s), excess liquid from the blot was dried, and the dried blot was exposed to a Storage Phosphor Screen (Amersham) overnight at room temperature and imaged using a Typhoon FLA9500 scanner (GE Healthcare).

### Bioinformatic analyses

To computationally search for FtsH proteins across phyla, PaFtsH1 (accession number: EWH24232.1) and PaFtsH2 (accession number: EWH27927.1) of *P. aeruginosa* SG17M were used as queries to identify homologs in the NCBI and UniProt databases by BlastP using standard parameters (64, 65). Due to the high conservation of FtsH, standard search parameters (word size: 5; matrix; BLOSUM62; gap costs: existence 11, extension 1) allow the identification of FtsH proteases in all branches of life including *H. sapiens* and the plant *A. thaliana.* Selection criteria for searches with PaFtsH1 and PaFtsH2 to build the phylogenetic tree were >93% coverage over the entire length of the amino acid sequence to account for the sequence diversity at the N- and C-termini of the protein and to retrieve only full-length proteins combined with a identity threshold of > 50%. This threshold was chosen to avoid overlap between the PaFtsH1 and PaFtsH2 groups, while different empirical thresholds were chosen for the analysis of linker sequences in protein subgroups. Identical protein sequences were subsequently removed. In addition, representative FtsH proteins, with the consideration of one representative protein per genus and up to three proteins per phylum, from all major bacterial phyla; selected FtsH protein sequences from UniProt; representative proteins from other branches of the phylogenetic tree including plant FtsH representatives from *A*. *thaliana* and mammalian representatives from *H. sapiens* were included in the assessment of phylogenetic relatedness. Subsequently, proteins were aligned using ClustalX2 using standard parameters and manually curated (66). The aligned sequences were subjected to phylogenetic analysis using preliminary neighbor-joining and conclusively maximum likelihood in MEGA7.0 or MEGA 10.2.2 (67). The Poisson model was used as an amino acid replacement model. The robustness of the phylogenetic tree topologies was evaluated by bootstrap analysis with 100 replications. The trees were displayed with MEGA or iTOL (68).

To analyze the linker sequences, the protein alignment was inspected manually in ClustalX2 (69) and GeneDoc (nrbsc.org/gfx/genedoc). Subsequently, considering the overall protein identity, proteins with linker sequences most homologous to the PaFtsH1 and PaFtsH2 linker sequence were selected. Only few linkers of PaFtsH1-like proteases were omitted due to substantial evolution such as missing the highly conserved R(Q/R/K)MQ motif as in NLA45192.1 (RGVS) and OGN14899.1 (RMTT) or missing the linker at all (Fig. 5*A*, indicated in black). WebLogos which display the frequency of amino acid occurrence at one position were created using the resulting linker sequence alignments for typical PaFtsH1-like and PaFtsH2-like linkers (70). Linker sequences for alternative PaFtsH1 and PaFtsH2 related subgroups were retrieved in a similar approach.

Structural prediction of the MC linker in Figure 5*C* was calculated using PEP-FOLD 3.5 (71). Structural prediction of *P. aeruginosa* RpoH (PaRpoH) was calculated using ROSETTA (72). PaFtsH1 and PaFtsH2 monomer structures were predicted by AlphaFold (73) using accession numbers Q9HV38_PSEAE for PaFtsH1 and A0485GIM3_PSEI for PaFtsH2. The following residue changes were made for A0485GIM3_PSEI to achieve 100% sequence identity with PaFtsH2: G68S, I447M, and A524E in PyMOL 2.5.4 (The PyMOL Molecular Graphics System, Version 2.0 Schrödinger, LLC). Estimations of structural disorder were calculated by the IUPred algorithm (74). Charge *versus* hydropathy calculations were computed using PONDR protein disorder predict (75, 76, 77).

Transmembrane helices in PaFtsH1 and PaFtsH2 were evaluated by AlphaFold structural models, TOPCON and ΔG prediction server v1.0 (78, 79). Current and previous analysis was based on TMHMM 2.0, SOSUI and DAS (5).

### Electron microscopy and image processing

Samples were diluted to 0.4 mg/ml with storage buffer (50 mM Tris-HCl (pH 8.0), 10 mM KCl, 5 mM MgSO4, 10 μM ZnCl_2_, 60 mM imidazole, 10% glycerol, 0.1% Igepal and 2 mM β-mercaptoethanol). 3.0 μl of the dilutions were placed to glow-discharged copper grids with continuous carbon support film and incubated for 1 min. Excess sample was blotted away with filter paper and the grids washed in three drops of Milli-Q water before staining 30 s in 2% uranyl acetate (TAAB Laboratories Equipment Ltd). After most stain was blotted away with the filter paper, the grids were let to air-dry. Samples were checked in JEM-2100f field emission electron microscope (JEOL Ltd) operated at 200 kV. Images were collected at 50kx nominal magnification and 0.7 to 1.5 μm defocus with Tvips TemCam XF416-camera (Tietz Video and Image Processing Systems GmbH) using real-time drift correction.

All image processing was done in EMAN2 (version 2.2; (80). Particles were boxed in e2boxer_old using swarm mode using box size of 128 pixels. The extracted particles were corrected for the contrast transfer function. 2D classifications were used for estimating data quality and resulting 2D classes to create initial models both without symmetry and with the proposed C6-symmetry. Initial models were further refined during following 3D refinement rounds. Final particle sets for 3D-reconstructions in e2refine_easy using C6 symmetry and defined target resolution 18.0 Å contained 4781 (PaFtsH1), 2921 (PaFtsH2) and 3510 (PaFtsH2^H1-link-32^) particles. Docking of atomic structures was achieved using UCSF Chimera (81).

Structures were deposited in wwPDB EMDB database with accession numbers EMD-18387 (Deposition ID D_1292133141; PaFtsH1), EMD-18388 (Deposition ID D_1292133170; PaFtsH2) and EMD-18389 (Deposition ID D_1292133186; PaFtsH2^H1-link32^).

## Data availability

All material, strains, and plasmids are available from either Tania A. Baker (tabaker@mit.edu) or Ute Römling (Ute.Romling@ki.se). pJN105- and pET-28a(+)-based plasmids with cloned Pa*ftsH1*, Pa*ftsH2*, Pa*ftsH1*^*NFtsH2*^, Pa*ftsH2*^*NFtsH1*^ and Pa*ftsH2*^*H1-link-32*^ have been submitted to Addgene.

## Supporting information

This article contains supporting information (82, 83, 84). A PDF file with supporting information containing SI Figures S1-S11 and an Excel file with SI Tables S1-S4 are provided.

## Conflicts of interest

The authors declare that they have no conflicts of interest with the contents of this article.

## Supporting information

Supplementary Tables1-4
