# Supplementary material for: The membrane-cytoplasmic linker defines activity of FtsH proteases in Pseudomonas aeruginosa clone C
Source: J Biol Chem. 2024 Jan 3;300(2):105622. doi: 10.1016/j.jbc.2023.105622 (PMC10850787; doi:10.1016/j.jbc.2023.105622)
Supplement: Supporting information [file mmc2.pdf]

# Supporting Information

## The membrane-cytoplasmic linker defines activity of FtsH proteases in *Pseudomonas aeruginosa* clone C

[1&]Mawla, Gina D.; [2]Kamal, Shady M.; [2]Cao, Lian-Ying; [3]Purhonen, Pasi; [3]Hebert, Hans; [1]Sauer, Robert T.; [*1#]Baker, Tania A.; and [*2#]Römling, Ute

[1]Department of Biology, Massachusetts Institute of Technology, Cambridge, MA; USA

[2]Department of Microbiology, Tumor and Cell Biology, Karolinska Institutet, Stockholm; Sweden

[3]Department of Biomedical Engineering and Health Systems, KTH Royal Institute of Technology, Huddinge; Sweden

[&]Current address: Division of Biology and Biological Engineering, California Institute of Technology, Pasadena, California 91125, USA

*Running title: divergent FtsH protease functionality*

Keywords: cytoplasmic linker, essential protease, *Pseudomonas aeruginosa* clone C, ssrA-tag; AAA+ ATPase; M41 protease: periplasmic domain

#contributed equally
* Corresponding Author: tabaker@mit.edu and Ute.Romling@ki.se

## Supporting Information – Content

## FIGURES

**Supporting Information Figure 1. Sequence alignment of FtsH homologs.**
**Supporting Information Figure 2. FtsH enzymes from *P. aeruginosa* SG17M clone C are located at distinct genetic loci and exhibit different temperature-dependent ATPase rates.**
**Supporting Information Figure 3. Degradation of $\lambda cI^{N}$-ssrA by PaFtsH2 MC linker variants requires the presence of an ssrA degron, ATP, and proceeds in the C-to-N direction.**
**Supporting Information Figure 4. Binding of radioactively labelled PaFtsH1 and PaFtsH2 on peptide arrays of potential and verified substrates.**
**Supporting Information Figure 5. Purification profile of the linker variant PaFtsH2$^{H1\text{-}link\text{-}32}$ and PaFtsH2 MC linker variants display enhanced disorder in the MC linker region.**
**Supporting Information Figure 6. Negative-stain TEM structures of PaFtsH1, PaFtsH2 and PaFtsH2$^{H1\text{-}link\text{-}32}$.**
**Supporting Information Figure 7. Assessment of zone of inhibition upon treatment with the aminoglycoside antibiotic tobramycin.**
**Supporting Information Figure 8. Linker evolution in closely related PaFtsH1 and PaFtsH2 homologs as displayed by WebLogos.**
**Supporting Information Figure 9. FtsH linker evolution in deeply branching bacteria.**
**Supporting Information Figure 10. ATPase activity and growth complementation of PaFtsH2 AAA and GGG insertion variants.**
**Supporting Information Figure 11. Phylogenetic analyses of FtsH homologs from phyla carrying supernumerary copies of FtsH.**

**TABLES**

**Supporting Information Table 1. Peptide array sequences.**
**Supporting Information Table 2. List of FtsH proteins used to construct the phylogenetic tree in Fig. 5A.**
**Supporting Information Table 3. Plasmids used in this study.**
**Supporting Information Table 4. Primers used in this study.**

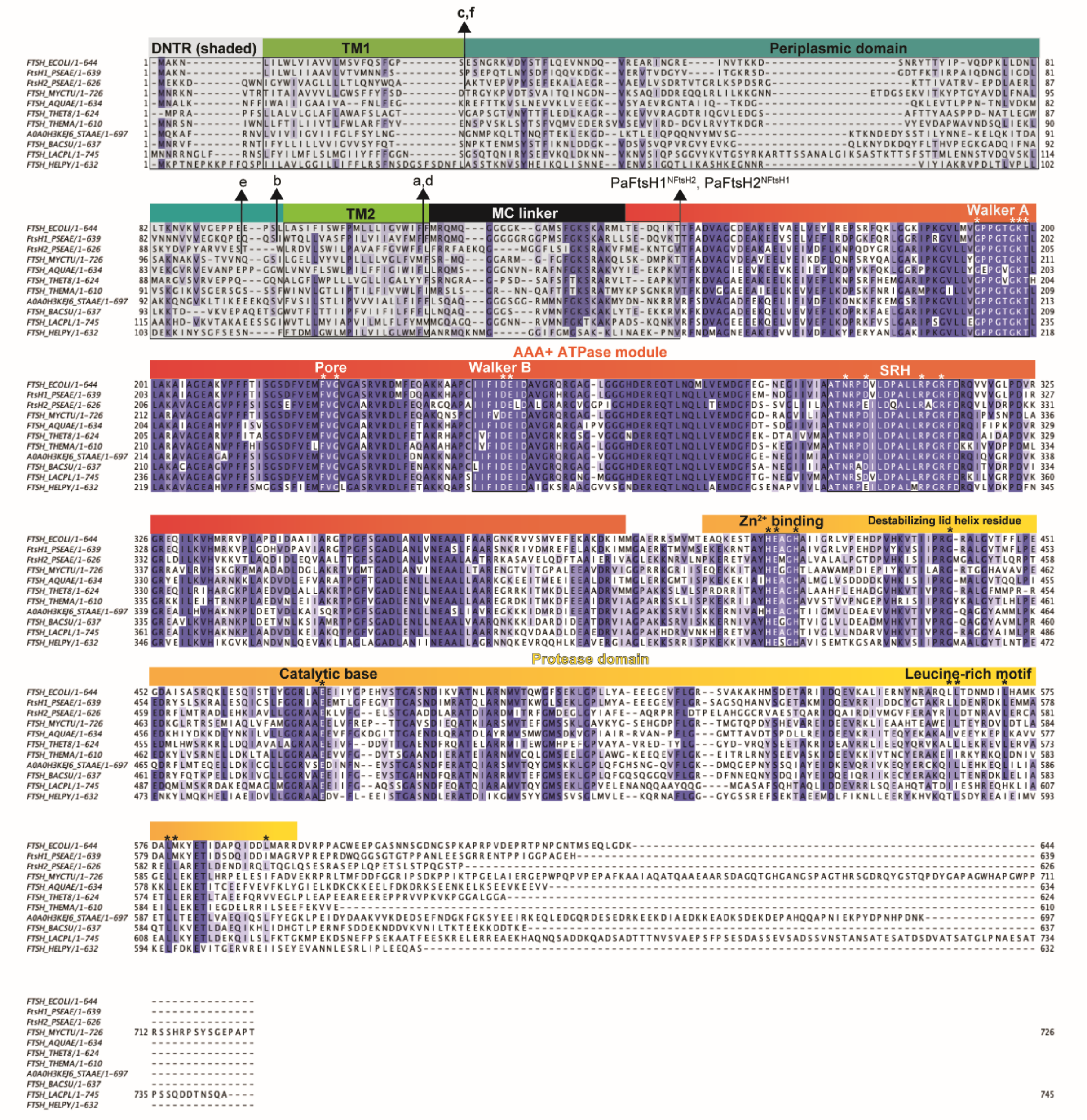

**SI Figure 1: Sequence alignment of FtsH homologs.** The multiple sequence alignment was constructed based on the full-length protein sequences of FtsH from *Escherichia coli*, *Pseudomonas aeruginosa*, *Mycobacterium tuberculosis*, *Aquifex aeolicus*, *Thermus thermophilus*, *Thermus maritima*, *Staphylococcus aureus* (Newman strain), *Bacillus subtilis*, *Lactiplantibacillus plantarum*, and *Helicobacter pylori*. Sequences comprising the diverse N-terminal region (DNTR) are boxed and shaded in grey (residues 1-149 in *E. coli* FtsH) and include transmembrane domains 1 and 2 (TM1 and TM2), the periplasmic domain, the membrane-cytoplasmic (MC) linker, and part of the first beta sheet in the first AAA+ ATPase module (corresponding to residues 143-149 in *E. coli* FtsH). Protein domains and key residues (star) are indicated in the colored bars directly above the multiple sequence alignment. Conserved between PaFtsH1 and PaFtsH2 are the FVG pore motif and the ATP-binding Walker A and Walker B motifs in the AAA+ ATPase module; and the $Zn^{2+}$ binding motif, the destabilizing lid helix residue, the catalytic base and the leucine-rich motif in the M41 protease domain. Arrows and letters refer to the border of the hybrid constructs described in Figure 2D and E.

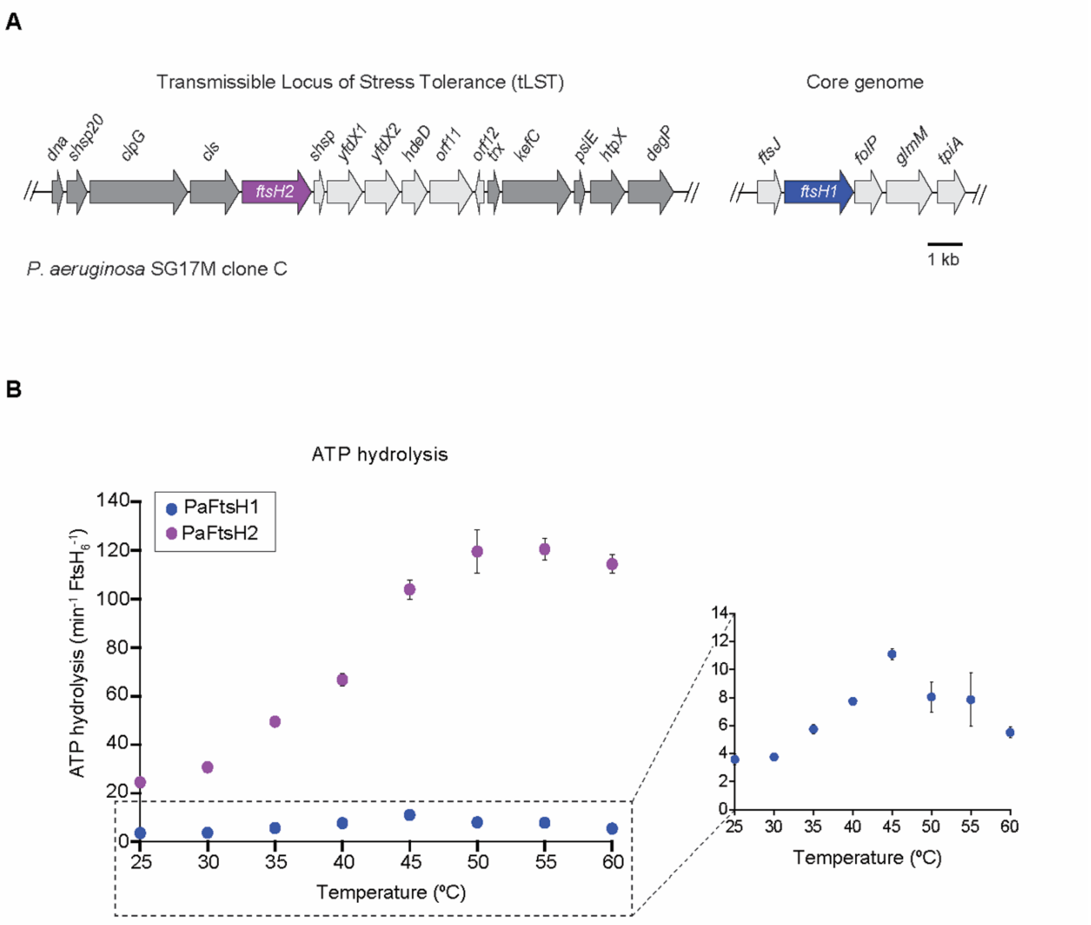


**SI Figure 2. Genes encoding PaFtsH proteases are found at distinct chromosomal locations in *P. aeruginosa* SG17M clone C and the enzymes exhibit different temperature-dependent ATPase rates.** A) Genetic loci of Pa*ftsH* genes in *P. aeruginosa* SG17M clone C strain. Note that PaFtsH1 is encoded on the core genome of SG17M, and PaFtsH2 is encoded on a horizontally-acquired genomic island termed the transmissible locus of stress tolerance (tLST) distinctive of clone C strains including its representative SG17M. Dark grey, tLST genes of SG17M; light grey, tLST genes found in the full-length tLST island. B) Hydrolysis of 5 mM ATP by 0.4 µM PaFtsH1 or PaFtsH2 at temperatures ranging from 25°C-55°C. Data points are three independent replicates performed in duplicate ± SD.

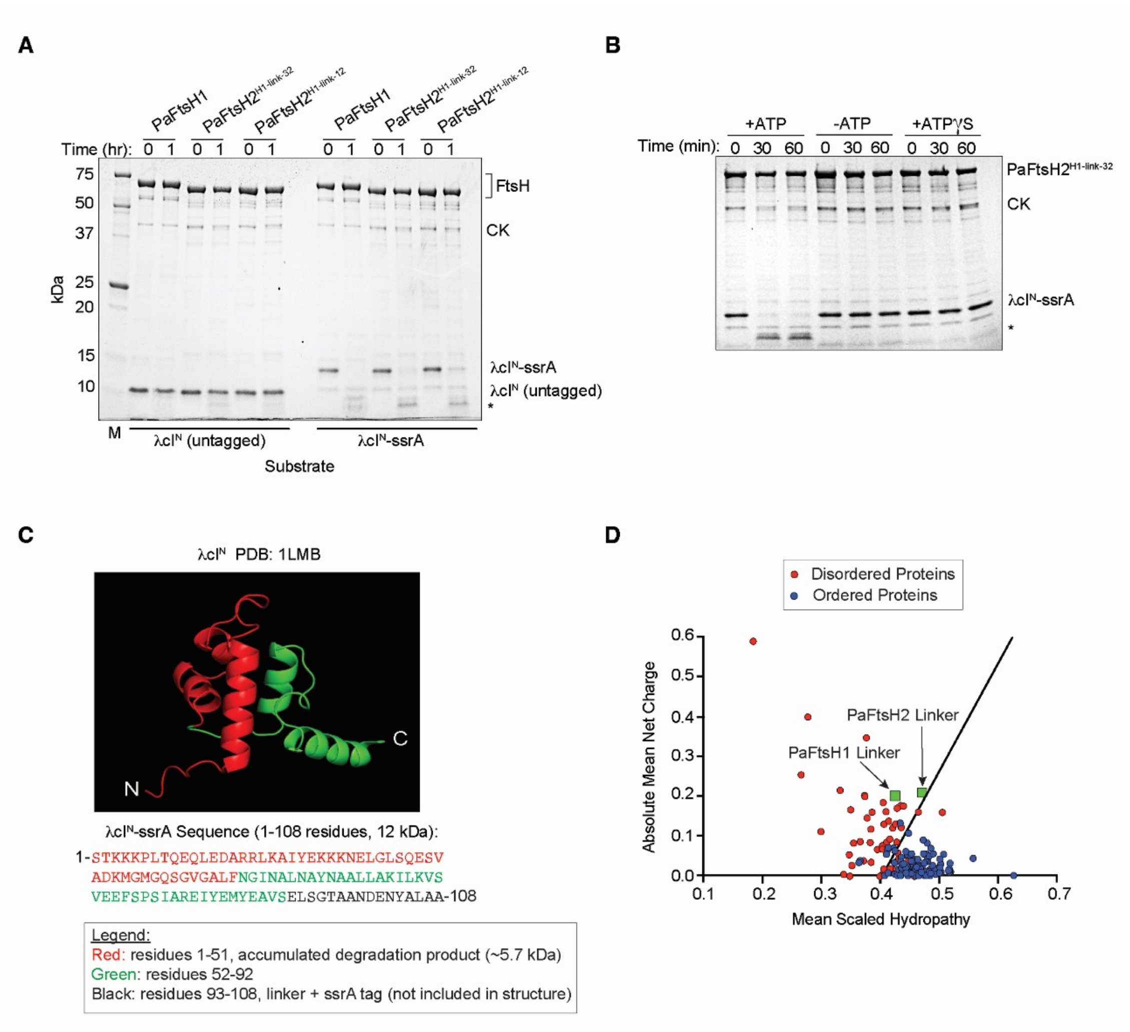


**SI Figure 3. Degradation of λcI$^{N}$-ssrA by PaFtsH2 MC linker variants requires the presence of an ssrA degron, ATP, and proceeds in the C-to-N direction.** A) Degradation of untagged λcI$^{N}$ (15 µM) or λcI$^{N}$-ssrA (15 µM) by PaFtsH1, PaFtsH2$^{H1\text{-}link\text{-}32}$ or PaFtsH2$^{H1\text{-}link\text{-}12}$ (3.04 µM hexamer equivalents) at 40ºC in the presence of 5 mM ATP and a creatine kinase (CK) based ATP regeneration system after one hour. The reaction has been monitored by a 4%-20% gradient SDS-PAGE protein gel. Bands corresponding to full-length λcI$^{N}$ (~10 kDa) and λcI$^{N}$-ssrA substrates (~14 kDa) are indicated on the gel. Asterisk indicates a ~5.7 kDa degradation intermediate described in panel (B). Reactions containing PaFtsH1 served as a positive control for ssrA tag-dependent proteolysis. B) ATP hydrolysis is required for efficient proteolysis of λcI$^{N}$-ssrA by PaFtsH2$^{H1\text{-}link\text{-}32}$. Experimental conditions are the same as (A) except reactions either contained 5 mM ATP, 0 mM ATP, or 5 mM ATPγS. Asterisk indicates a ~5.7 kDa degradation intermediate in lanes of the 30- and 60-minute time points +ATP reactions. C) The ~5.7 kDa, λcI$^{N}$-ssrA degradation intermediate product that accumulates in proteolysis reactions shown in (A) and (B) corresponds to N-terminal residues 1-51 of λcI$^{N}$. Structure of λcI$^{N}$ (top; PDB: 1LMB (83)) and primary sequence (bottom). Residues 1-51 are labeled in red on both the sequence and structure have been detected by LC-MS/MS. D) Charge-versus-hydropathy plot of PaFtsH1 MC linker (amino-acids 120-144) and PaFtsH2 MC linker (amino acids 124-147) against a reference set of disordered (red points) and ordered (blue points) proteins as calculated by PONDR® (see Experimental Procedures). Solid black line indicates the measured hydropathy boundary between the reference set of disordered

and ordered proteins. PaFtsH1 MC linker (mean scaled hydropathy: 0.4253, absolute mean net charge: 0.2); PaFtsH2 MC linker (0.4713, 0.2083).

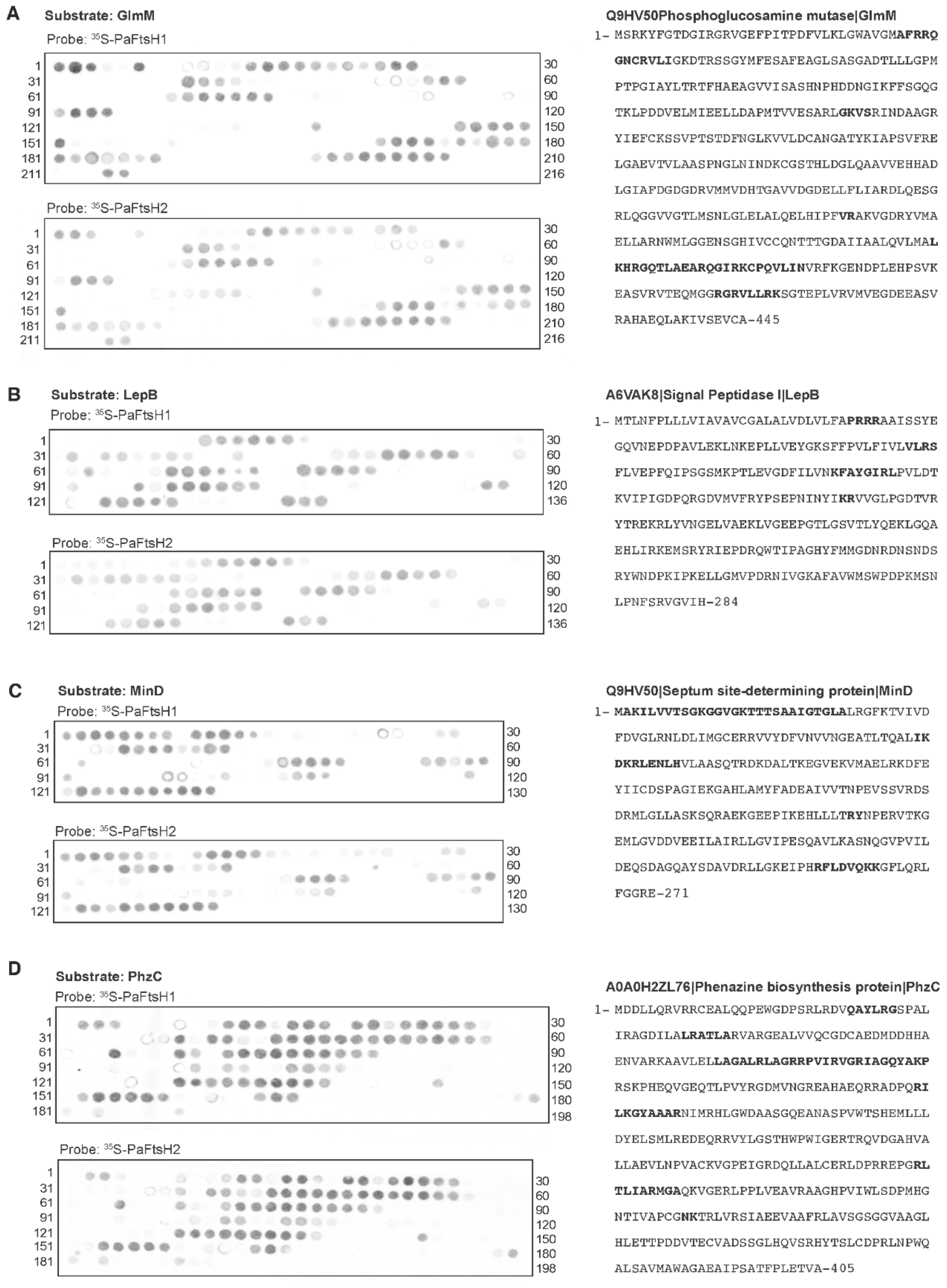


**SI Figure 4. Binding of radioactively labelled PaFtsH1 and PaFtsH2 to peptide arrays of potential and verified substrates.** A-D) PaFtsH1 and PaFtsH2 binding to peptide arrays of three putative and one verified PaFtsH1/PaFtsH2 substrate as identified by Kamal et al, 2019 ((5); left panels) along with their amino acid sequences (right panels). PhzC1/2 has been identified as a PaFtsH1 substrate, GlmM and MinD are putative PaFtsH1 substrates and LepB1 is a putative PaFtsH2 substrate. Peptide arrays contain

spots mapping to a sliding window of 12 amino acids with a step size of two amino-acids towards the C-terminus with each spot (from left to right) and were probed with 1 μM $^{35}$S-PaFtsH1 or $^{35}$S-PaFtsH2 in the presence of 1.25 mM ATPγS. Full-length protein sequences corresponding to the peptide arrays are indicated in the panels on the right of each figure. Residues in bold indicate regions bound by $^{35}$S-PaFtsH1 or $^{35}$S-PaFtsH2. Peptide sequences corresponding to each spot on the arrays are listed in Table S1. Substrates names and accession IDs in (A-D) are as follows: A) Phosphoglucosamine mutase (GlmM; Q9HV50|GlmM_PSEAE). B) Signal peptidase I (LepB1; A6VAK8|LepB_PSEA7). C) Septum site-determining protein (MinD; K0E51_10040|MinD_PSEAE_SG17M Assembly GCF020978345.1). D) Phospho-2-dehydro-3-deoxyheptonate aldolase (PhzC1/2 (identical); A0A0H2ZL76|PhzC2_PSEAB).

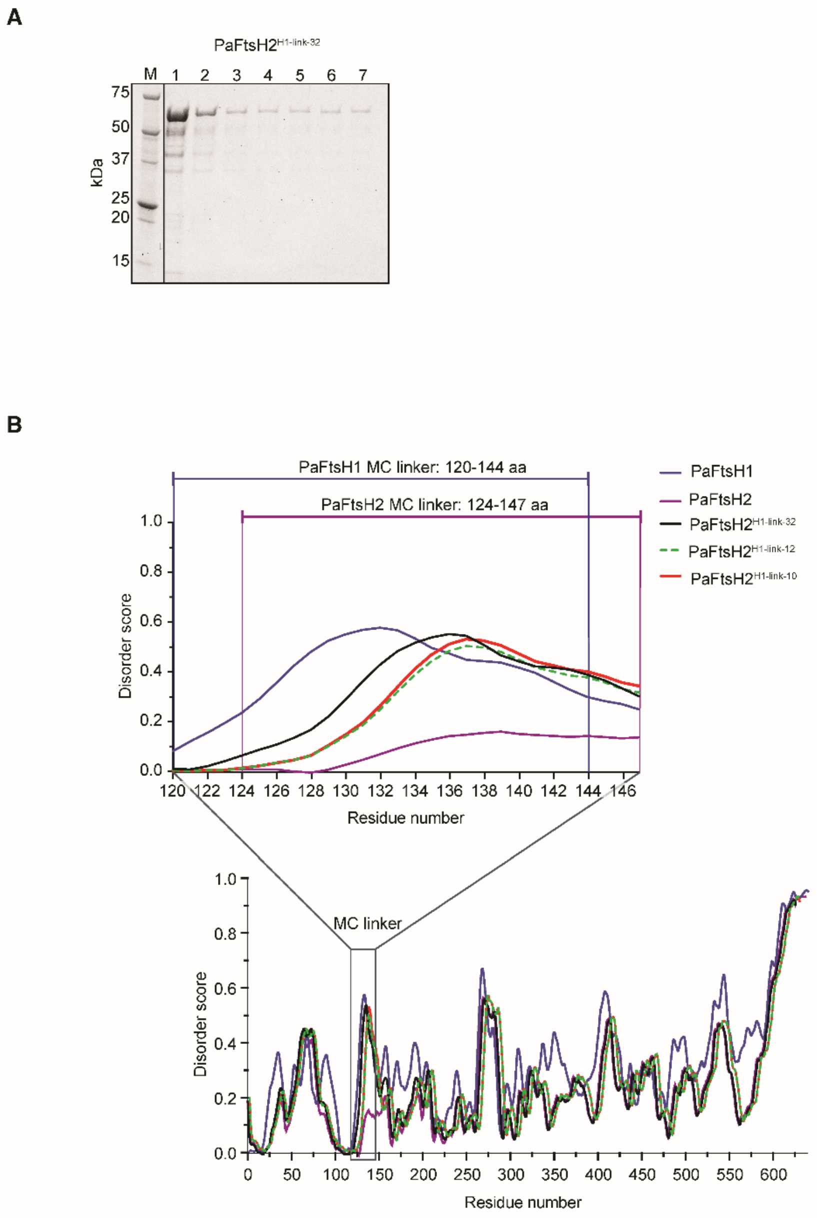


**SI Figure 5. Purification profile of the linker variant PaFtsH2$^{H1\text{-}link\text{-}32}$ and PaFtsH2 MC linker variants display enhanced disorder in the MC linker region**. A) Exemplary demonstration of elution fractions of purified PaFtsH$^{H1\text{-}link\text{-}32}$ separated on a 4%-20% gradient SDS-PAGE protein gel and stained with Coomassie brilliant blue R-250. M: Molecular weight marker. Molecular weight of PaFtsH$^{H1\text{-}link\text{-}32}$ is 69288.2 Da. B) Traces are predicted structural disorder profiles (74) of PaFtsH2 MC linker variants in relation to wild type PaFtsH2. Enlarged boxed region displays the values for MC linker residues. Predicted disorder increases with increasing score.

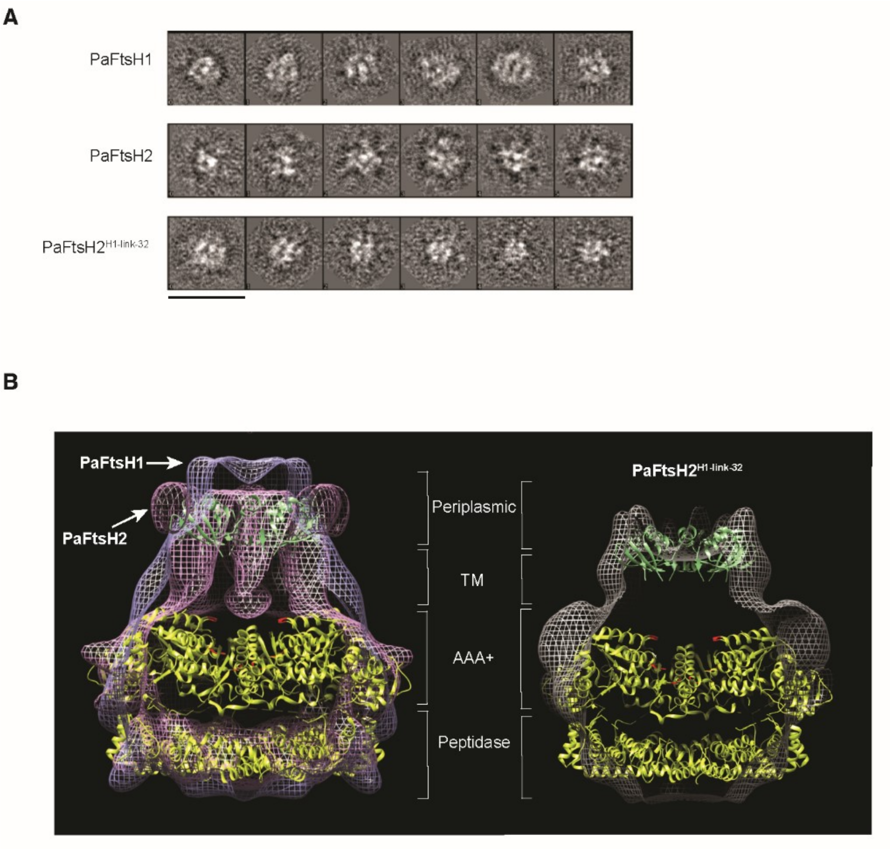


**SI Figure 6. Negative-stain TEM structures of PaFtsH1, PaFtsH2 and PaFtsH2$^{\text{H1-link-32}}$.** A) Negative-stain TEM images of 2D class average single particles of PaFtsH1 (top), PaFtsH2 (middle), and PaFtsH2$^{\text{H1-link-32}}$ (bottom). Box size and scale bar are 28 nm. B) Cross-sections of 3D reconstruction TEM density maps of PaFtsH1 (blue) and PaFtsH2 (purple) on the left. Cross-section of 3D electron microscopy density map of PaFtsH2$^{\text{H1-link-32}}$ (grey; right). The FtsH cytoplasmic domain from *A. aeolicus* (yellow; PDB: 4WW0, (41)) and periplasmic domain from *E. coli* (green; PDB: 4V0B, (84)) were docked onto the density map of PaFtsH2. FVG pore loops are colored in red. Abbreviations are as follows: Periplasmic: Periplasmic domain; TM: Transmembrane domain; AAA+: AAA+ unfoldase domain; Peptidase, M41 proteinase domain.

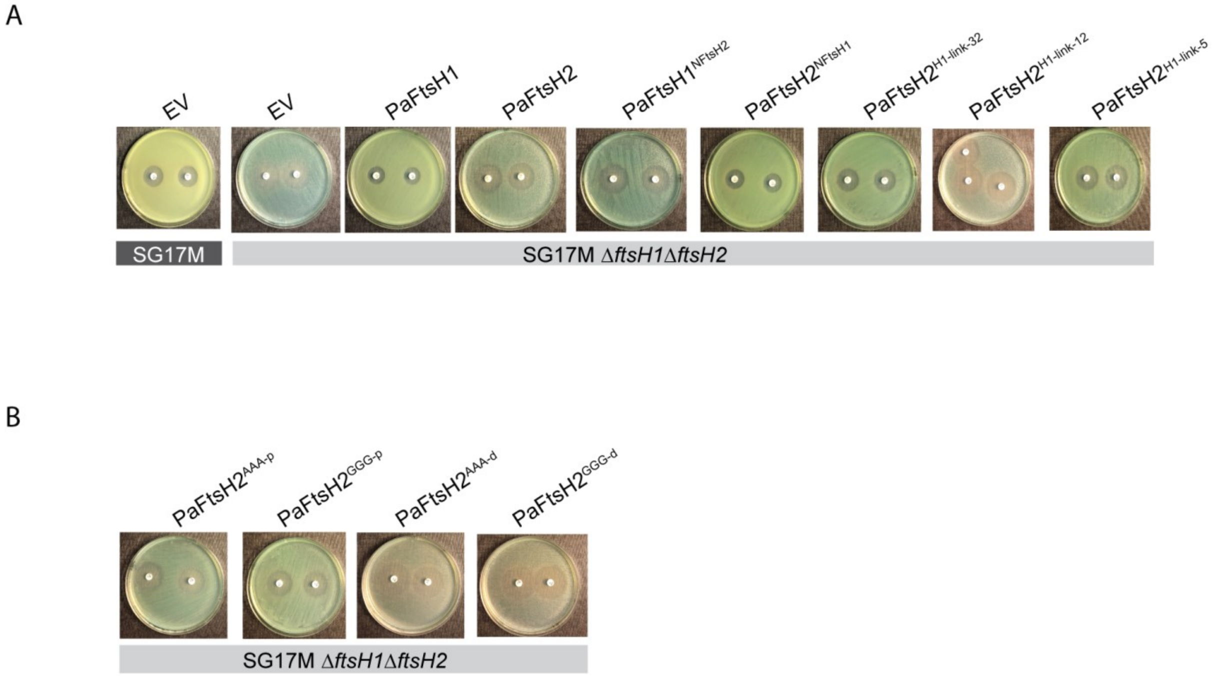


**SI Figure 7. Assessment of zone of inhibition for *P aeruginosa* SG17M, SG17M Δ*ftsH1*Δ*ftsH2* and complemented strains upon treatment with the aminoglycoside antibiotic tobramycin.** A) *P. aeruginosa* SG17M Δ*ftsH1*Δ*ftsH2* double deletion strain was complemented by expression of PaFtsH1 and PaFtsH2 wild type and diverse N-terminal region (DNTR) and linker variants as indicated. Cell suspensions were streaked out evenly on Mueller Hinton agar plates to which two tobramycin discs (10 µg) were subsequently placed. The tobramycin-disc-containing plate was incubated at 37ºC and the diameter of the inhibition zone was measured after 20-24 h. Quantification of zone of inhibition is shown in Figure 4B. Reference *P. aeruginosa* SG17M; EV: Empty vector control pJN105. B) Same as in A), but with PaFtsH2 triple amino acid insertion variants.

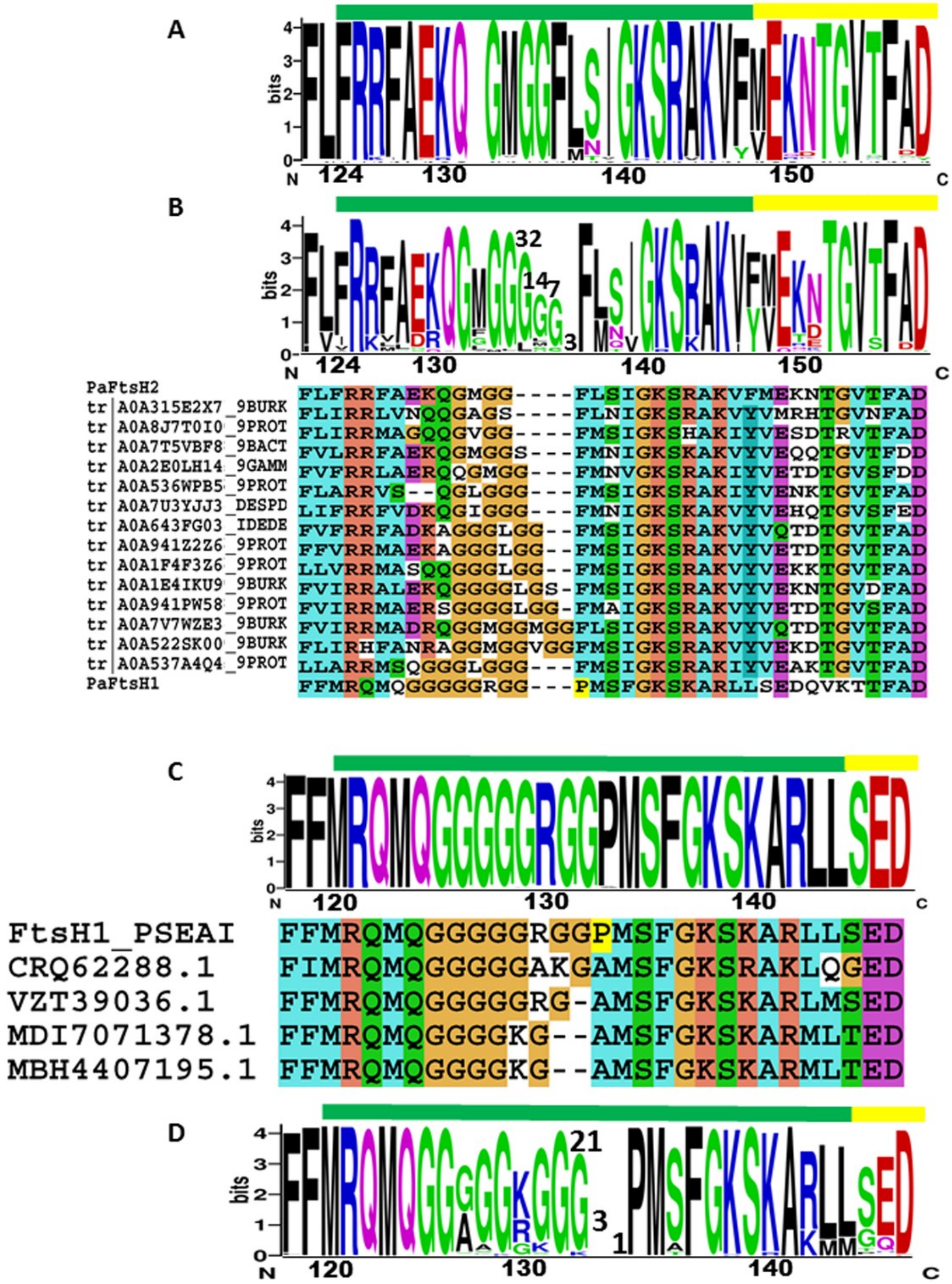


**SI Figure 8. Linker evolution in closely related PaFtsH1 and PaFtsH2 homologs as displayed by WebLogos.** A) WebLogo of the linker sequences of PaFtsH2 homologs with an identity >80% as derived from the UniProt and NCBI database. Of 287 protein sequences from different species, only one FtsH2 homolog showed a longer linker with the sequence QQGMGG. B) WebLogo of the linker sequences of PaFtsH2 homologs with >67.5% identity as derived from the UniProt and NCBI database. Of 252 protein sequences from different species, 32 protein sequences showed a longer linker containing additional glycine residues. C) WebLogo of the linker sequences of PaFtsH1 proteins in *P. aeruginosa* with an identity >50% (query coverage >90%) retrieved by BLAST from the NCBI database. Of 252 non-redundant PaFtsH1 sequences with an identity >50%, four PaFtsH1 proteins showed a reduction of the glycine content and/or shorter linker. D) WebLogo of linker sequences of PaFtsH1 homologs from the UniProt and NCBI database. 21 of 248 proteins showed a longer linker, while only one protein

showed a reduced linker with the sequence MQGGGGGKGP. Amino acid numbering as in PaFtsH1 and PaFtsH2. Green bar above WebLogo sequences indicates MC linker; yellow bar indicates start of AAA+ ATPase module.

A

B

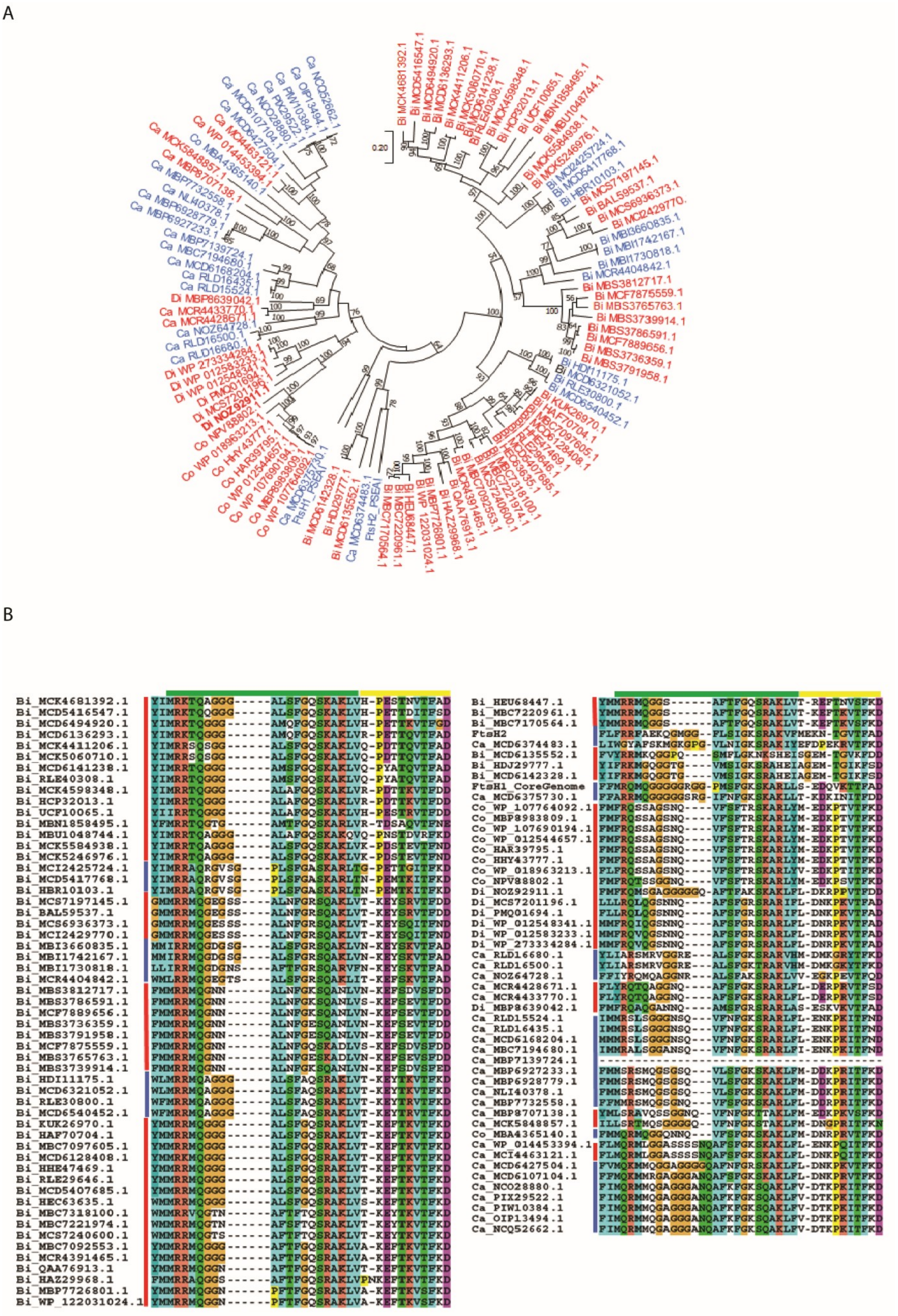

**SI Figure 9. FtsH linker evolution in selected deeply branching bacterial phyla.** All FtsH sequences were retrieved by standard protein-protein BLAST search from the NCBI database from deeply branching phyla Bipolaricaulota (Bi), Caldiserica (Ca), Dictyoglomota (Di) and Coprothermobacterota (Co) were aligned and the linker sequences were analyzed. A) Maximum-likelihood based phylogenetic tree of the FtsH proteins constructed and displayed in MEGA 7.0. B) Aligned linker sequences. Green bar above sequence alignment indicates MC linker sequence; yellow bar: +7 sequence of the MC linker sequence constituted by the first β-strand of the AAA+ ATPase module. Red and blue bars indicate different classes of linker sequences which correspond to FtsH protein subgroup designations in the phylogenetic tree.

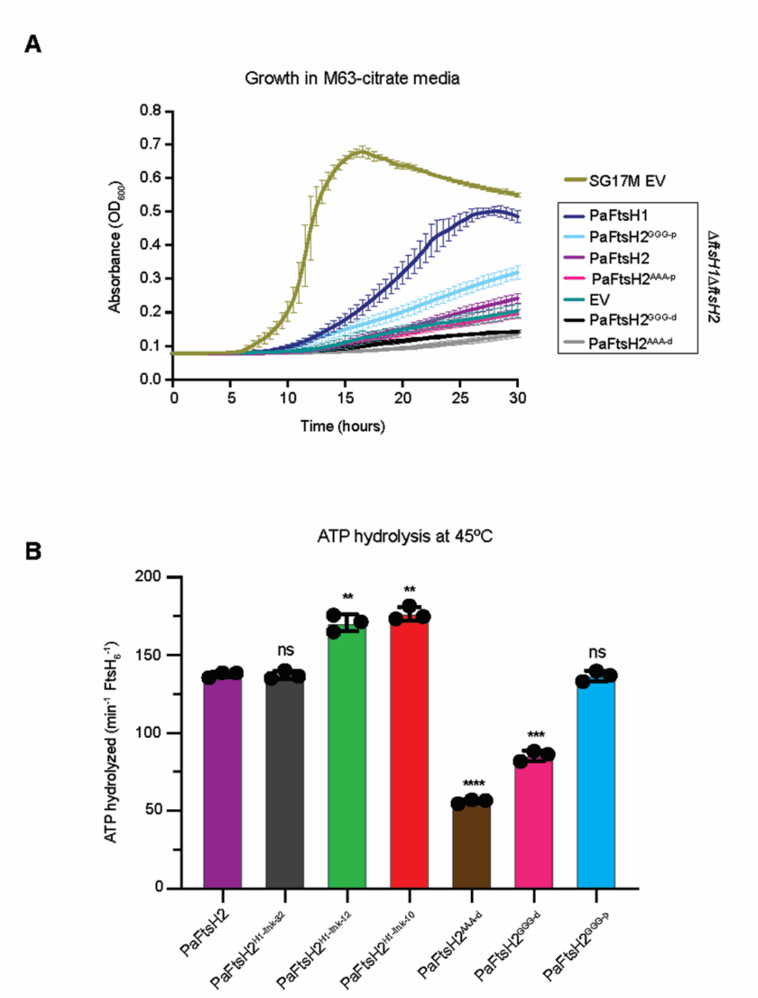


**SI Figure 10. Growth complementation by PaFtsH2 GGG and AAA insertion variants and ATPase activity of PaFtsH2 proteins with hybrid linker.** A) Growth curves of *P. aeruginosa* SG17M, *P. aeruginosa* SG17M Δ*ftsH1*Δ*ftsH2* double deletion and *P. aeruginosa* SG17M Δ*ftsH1*Δ*ftsH2* double deletion complemented with PaFtsH2 constructs with distal and proximal GGG and AAA insertions in the MC linker in M63-citrate medium. The *P. aeruginosa* SG17M Δ*ftsH1*Δ*ftsH2* double deletion strain had been complemented with wild type PaFtsH1 and PaFtsH2 as a reference. EV: Empty vector control pJN105. B) Hydrolysis of ATP (5 mM) by PaFtsH2 (0.4 µM) variants at 45ºC. Data points are three independent replicates performed in duplicate ± SD. **** $P < 0.00002$; *** $P < 0.0002$; ** $P < 0.002$; ns: not significant (compared to wild type PaFtsH2 (purple bar)) as analyzed by an unpaired t-test with Welch's correction.

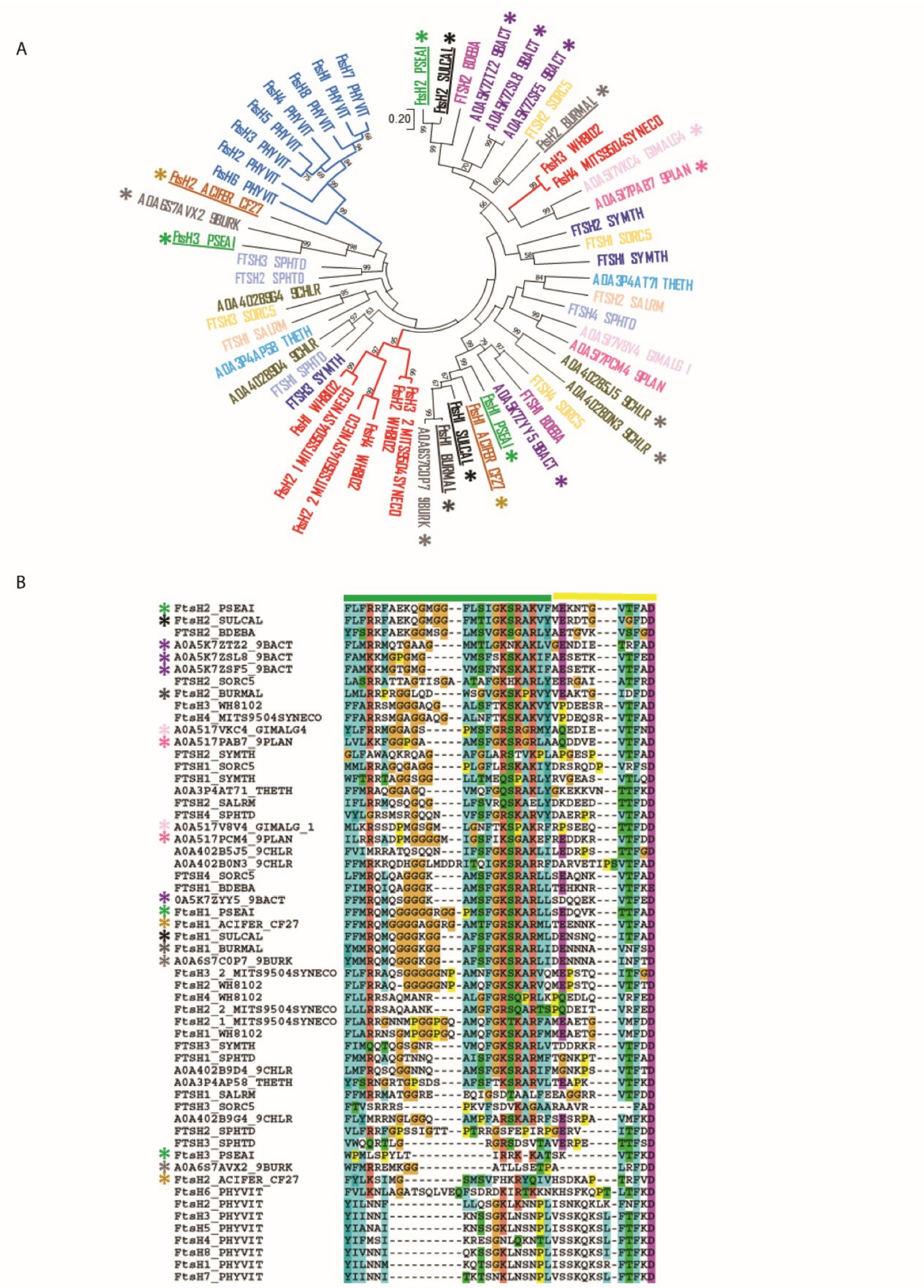


**SI Figure 11. Phylogenetic analyses and linker sequences of FtsH homologs from phyla carrying supernumerary copies of FtsH.** A) Phylogenetic tree with FtsH proteins of representative strains of species encoding supernumerary copies of FtsH proteins. Underlined sequences are also included as references in Figure 5A. B) Aligned linker sequences of FtsH proteins from the phylogenetic tree in A). Green bar above sequence alignment indicates MC linker sequence; yellow bar: +7 sequence of the MC linker sequence constituting the first β-strand of the AAA+ ATPase module. Stars highlight FtsH proteins from selected bacterial isolates (color code as in A)). In green, PaFtsH proteins found in *P.*

*aeruginosa*. In red, FtsH proteins of *Synechococcus* species. In dark blue, FtsH proteins of phytoplasma *Flavescence doree.* In yellow, from *Sorangium cellulosum*. In light blue, from *Sphaerobacter thermophilus*. In grey, from *Dictyobacter alpinus*. In dark pink, from *Bdellovibrio bacteriovorus.* In blue, from *Thermus thermophilus.* In skin colour, from *Salinibacter ruber*. In dark violet, from *Symbiobacterium thermophilum.* In violet, from *Desulfosarcina ovata* subsp. *sediminis*. In light pink, from *Gimesia algae.* In pink, from *Alienimonas californiensis*. In brown, from *Acidithiobacillus ferrivorans*. In light grey, from *Paraburkholderia ultramafica*. In dark grey, from *Burkholderia mallei*. In black, from *Sulfuritortus calidifontis.*

Species with multiple FtsH proteases were derived from the following phyla: Mycoplasmatota: phytoplasma *Flavescence doree* FtsH6_PHYVIT, A0A2X0QB12, FtsH2_PHYVIT, A0A2X0QAH5, FtsH4_PHYVIT, A0A2X0QAI9, FtsH3_PHYVIT, A0A2X0QAN4, FtsH5_PHYVIT, A0A2X0RU37, FtsH7_PHYVIT, A0A2X0QAH5, FTSH8_PHYVIT, A0A2X0QW8, FtsH1_PHYVIT, A0A2X0QBI2. *Cyanobacteria: Synechococcus sp. MIT S9504: FtsH2* **1** MITS9504SYNECO, A0A164AW75; FtsH2 2 MITS9504SYNECO, A0A164AZH0; FtsH3 2 MITS9504SYNECO, A0A163YNW8; FtsH4 MITS9504SYNECO, A0A162CLW6. *Parasynechococcus marenigrum* WH8102:. FtsH1 WH8102, Q7U6N8, FtsH2 WH8102, A0A162CLW6, FtsH3 WH8102, Q7U5V4; FtsH4 WH8102, Q7U6X5. Myxococcota: *Sorangium cellulosum* (strain So ce56): FTSH1_SORC5, A9GRC9; FTSH4_SORC5, A9EXK6; FTSH2_SORC5, A9FDV9; FTSH3_SORC5, A9GAW6. Chloroflexota: *Dictyobacter alpinus*: A0A402B9G4_9CHLR, ftsH_6; A0A402B5J5_9CHLR, ftsH_3; A0A402B0N3_9CHLR, ftsH_2; A0A402B9D4_9CHLR, ftsH_5. Thermomicrobiota: *Sphaerobacter thermophilus* (strain DSM 20745): FTSH1_SPHTD, D1C1U7; FTSH2_SPHTD, D1C2C6; FTSH3_SPHTD, D1C4U5; FTSH4_SPHTD, D1C8C0. Bdellovibrionota: *Bdellovibrio bacteriovorus* (strain ATCC 15356): FTSH1_BDEBA, Q6MLS7; FTSH2_BDEBA, Q6MJV1. Deinococcota: *Thermus thermophilus*: A0A3P4AP58_THETH, ftsH_1; A0A3P4AT71_THETH, ftsH_2. Bacteroidetes: *Salinibacter ruber* (strain M8): FTSH1_SALRM, D5H7Z5; FTSH2_SALRM, D5HA94.1. Bacillota: *Symbiobacterium thermophilum* (strain T / IAM 14863): FTSH1_SYMTH, Q67LC0; FTSH2_SYMTH, Q67T82; FTSH3_SYMTH, Q67JH0. Thermosulfobacteriota: *Desulfosarcina ovata* subsp. *sediminis*: A0A5K7ZSF5_9BACT, ftsH_1; A0A5K7ZYY5_-9BACT, ftsH-2; A0A5K7ZSL8_9BACT, ftsH_2; A0A5K7ZTZ2_9BACT,ftsH_3. Planctomycetota: *Gimesia algae*: A0A517V8V4_GIMALG_1, ftsH_1; A0A517VKC4_-GIMALG4, ftsH4. *Alienimonas californiensis*: A0A517PAB7_9PLAN, ftsH4; A0A517PCM4_9PLAN, ftsH_8. Pseudomonadota: Acidithiobacilla: *Acidithiobacillus ferrivorans* CF27; FtsH1 ACIFER CF27, WP_035191373.1; FtsH2 ACIFER CF27, WP_051984798.1. γ-proteobacteria: FtsH1_PSEAI, FtsH2_PSEAI, PaFtsH3_PSEAI. β-proteobacteria: *Paraburkholderia ultramafica*: A0A6S7AVX2_9BURK, ftsH_1; A0A6S7-C0P7_9BURK, ftsH_4; *Burkholderia mallei* S13, FtsH1_BURMAL, WP_004191694.1; FtsH2_BURMAL, WP_004199606.1. *Sulfuritortus calidifontis* FtsH1_SULCAL WP_126463327.1; FtsH2_SULCAL, WP_126461415.1.
